# The Caenorhabditis chemoreceptor gene families

**DOI:** 10.1186/1741-7007-6-42

**Published:** 2008-10-06

**Authors:** James H Thomas, Hugh M Robertson

**Affiliations:** 1 Department of Genome Sciences, University of Washington, Seattle, WA, USA; 2 Department of Entomology, University of Illinois, Urbana-Champaign, IL, USA

## Abstract

**Background:**

Chemoreceptor proteins mediate the first step in the transduction of environmental chemical stimuli, defining the breadth of detection and conferring stimulus specificity. Animal genomes contain families of genes encoding chemoreceptors that mediate taste, olfaction, and pheromone responses. The size and diversity of these families reflect the biology of chemoperception in specific species.

**Results:**

Based on manual curation and sequence comparisons among putative G-protein-coupled chemoreceptor genes in the nematode *Caenorhabditis elegans*, we identified approximately 1300 genes and 400 pseudogenes in the 19 largest gene families, most of which fall into larger superfamilies. In the related species *C. briggsae *and *C. remanei*, we identified most or all genes in each of the 19 families. For most families, *C. elegans *has the largest number of genes and *C. briggsae *the smallest number, suggesting changes in the importance of chemoperception among the species. Protein trees reveal family-specific and species-specific patterns of gene duplication and gene loss. The frequency of strict orthologs varies among the families, from just over 50% in two families to less than 5% in three families. Several families include large species-specific expansions, mostly in *C. elegans *and *C. remanei*.

**Conclusion:**

Chemoreceptor gene families in *Caenorhabditis *species are large and evolutionarily dynamic as a result of gene duplication and gene loss. These dynamics shape the chemoreceptor gene complements in *Caenorhabditis *species and define the receptor space available for chemosensory responses. To explain these patterns, we propose the gray pawn hypothesis: individual genes are of little significance, but the aggregate of a large number of diverse genes is required to cover a large phenotype space.

## Background

Chemoperception is a central sense in nematodes, which lack vision and hearing. Detailed analysis of the cellular basis of chemosensory responses in *Caenorhabditis elegans *has shown that a small number of sensory neurons mediate all known responses [1]. Despite this cellular simplicity, a huge number of genes encode candidate receptors for chemical stimuli. The first indication of these genes came from an analysis of the partially completed genome sequence in 1995. This work identified several small genomic clusters of related genes encoding members of the rhodopsin superfamily of G-protein-coupled receptors (GPCRs), many of which were shown to be expressed in chemosensory neurons [2]. Subsequent analysis of the increasingly complete genome sequence identified additional large gene families with similar properties [3-7]. A brief summary of the families has been published as a book chapter [8]. Here we report a full analysis of all the largest putative GPCR chemoreceptor gene families, which comprise about 1700 genes and pseudogenes in the reference genome sequence, about 8.5% of all genes in *C. elegans*. Although direct evidence for a specific chemosensory function is limited to a single gene [9-11], expression patterns and other indirect evidence for many other members of the superfamily support a role predominantly in chemosensation [2,6,7,10]. For simplicity, we refer to these genes as chemosensory receptor or chemoreceptor genes, although some of the genes have probably acquired other functions (see, for example, [12]). A small number of other genes may also encode chemoreceptors, notably members of the TRP and guanylyl cyclase families [13-15]. These other families are unrelated in structure to GPCRs and are not included in our analysis.

Vertebrate chemoreceptor gene families have been the subject of intensive analysis. They mediate olfaction, taste, and pheromone responses and show remarkably plastic evolution at the level of gene duplication and gene loss (reviewed in [16,17]). For example, nearly the entire complement of vomeronasal receptor genes (thought to mediate pheromone responses) present in rodents has been deleted or pseudogenized on the primate lineage [18,19]. This genetic change is probably connected with increased emphasis on visual cues for social behaviors in primates [20]. *Drosophila melanogaster *has a surprisingly small number of chemoreceptor genes, including just 62 odorant and 68 gustatory receptor genes [21], which appear capable of mediating most of their known chemosensory responses (reviewed in [22-24]). Comparisons across 12 newly available *Drosophila *species genomes reveal that these insect chemoreceptors range in their evolutionary patterns from relatively conserved genes to relatively rapidly evolving gene lineages [25,26]. More distant comparisons with mosquitoes, moths, beetles, and bees reveal hugely expanded and contracted lineages over time frames of 250–300 My (million years) (see, for example, [27]). Remarkably, none of the known taste and olfactory receptors from insects and vertebrates have conserved homologs in *C. elegans*, suggesting either that they evolved independently or that they have diverged too much for the relationship to be recognizable.

Members of the *C. elegans *chemosensory receptor families form coherent sets of related sequences, falling into more than 20 families [3,4,6,7], of which the 19 largest are analyzed in this paper. A 20th family, srr, consists of only 10 genes in *C. elegans*, and may encode non-GPCR chemoreceptors (see [8] and HMR, unpublished). Within each chemoreceptor family, all of the genes share a common ancestor and the family has arisen through a long-term process of gene duplication, sequence divergence, and gene loss. The largest of the families is called srh, which includes about 310 loci in *C. elegans *[4]. These loci include genes that are probably functional, based on detailed gene annotation (~220 genes), and genes that appear to have nonfunctional alleles in the sequenced N2 genome (~90 genes). About half of the apparently nonfunctional alleles in N2 have only a single apparent defect (usually a stop codon, frameshift, or small deletion), and sequence analysis of several of these genes suggests that they have functional alleles in other wild isolates of *C. elegans *[28]. The other 18 chemoreceptor families range in size from 19 loci (srb and srxa families) to 267 loci (str family). All of the families have a substantial frequency of apparently nonfunctional genes in the N2 genome and show strong gene clustering in the genome, suggesting recent gene duplication [29].

As noted above for mammalian and insect chemoreceptors, a useful adjunct to studying a gene family in a specific organism is the capacity to compare the genes with those in related organisms with a variety of speciation dates. In this respect nematodes provide a relatively weak data set, because relatively few nematodes are under active study in the laboratory and the closest known relatives to *C. elegans *diverged as much as 100 My ago [30,31]. Nevertheless, the nearly complete sequences of two relatives, *C. briggsae *and *C. remanei*, are available, a draft assembly of *C. brenneri *is available, and sequencing projects are underway for several additional nematodes, ranging from other close *C. elegans *relatives to distant parasitic species [32-34]. For the sra, srab, and srz chemoreceptor gene families, published comparisons of *C. elegans *and *C. briggsae *indicate substantial differences in gene number and provide evidence of several expansions in gene number in *C. elegans *[6,7].

Here, we report an analysis of all 19 large chemoreceptor gene families in *C. elegans*, *C. briggsae *and *C. remanei*. We show that all of the families are characterized by substantial rates of birth-death evolution, with large variation in the rates in different families. We provide summaries of the evolution and conserved sequence features within each of the families.

## Results

### Curation of *C. elegans* chemoreceptor gene families

Genes encoding potential GPCR proteins were identified using a variety of methods (see Methods). Most of the GPCR gene candidates fell into families that are unrelated or distantly related to genes in other phyla. These predicted genes were used as a starting point to manually correct existing gene predictions and to identify a small number of new genes (see Methods). Among all of the predicted genes in a family, we identified probable full-length protein sequences and used these sequences as queries for *tblastn *searches to guide the correction of anomalous predictions. The large number of paralogs within each family made this approach effective. Candidate nonfunctional genes were identified as genes that could not be modified to give good full-length alignments. These included genes with in-frame stop codons, deletion of conserved parts of the family protein sequence, frameshifts in coding exons, probable splice junction defects, and missing start codons. The 19 largest gene families were chosen for further analysis. A complete list of encoded proteins for these families can be found in Additional file 1. Sample full-length protein alignments for each family are shown in Additional files 2 to 8. Alignments and analysis of nonfunctional genes were previously published for the srh and str families [3-6].

### *C. briggsae* and *C. remanei* annotation

Using computational methods we completed an improved annotation of genes from each of the 19 chemoreceptor families in *C. briggsae *and *C. remanei *(see Methods). Briefly, existing protein prediction sets for each species were combined with full-length *C. elegans *predictions and culled to identify all probable full-length proteins from the three species. The full-length protein set for each gene family was used in a *GeneWise *prediction pipeline, which was then combined with existing predictions to identify a best prediction for each gene. Subsequent analysis was based on these best prediction sets.

### Defective genes and gene family sizes

It was difficult to define accurately what should count as a functional gene or a defective gene in these families; the issues are described in detail in Methods. For *C. elegans*, the reference genome sequence is complete and our manual annotation was intensive enough to permit good estimates of the number of defective genes for each family, as summarized in Additional file 9. The frequency of defective genes was weakly correlated with family size; this issue is discussed further in the next section. Although it was clear that the reference *C. briggsae *and *C. remanei *genomes also include many defective genes, we did not accurately determine their number. Instead, genes were included for further analysis if they had the potential to encode nearly full-length proteins, as described in Methods. These gene counts are listed in Additional file 10, with *C. elegans *gene counts based on the same method. As some defective genes can encode near full-length proteins, the number of genes listed for *C. elegans *is larger than our estimate of the number of functional genes in Additional file 9. We presume that the number of functional genes in *C. briggsae *and *C. remanei *is similarly lower than the number of genes listed in Additional file 10. These near full-length protein predictions from the three species were used to construct protein trees; the protein sequences are given in Additional files 11, 12, and 13 (*C. elegans*, *C. briggsae*, and *C. remanei *respectively).

### Protein trees

**Figure 1:**
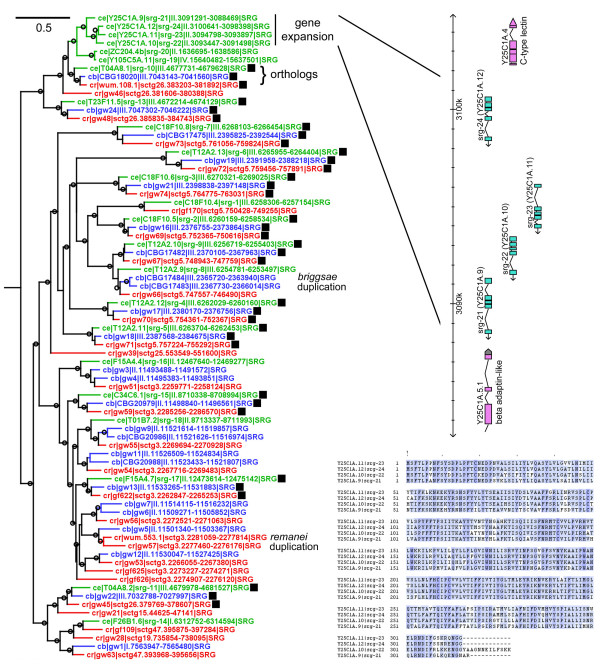
**Sample protein tree**. This tree is a section from the complete SRG protein tree (Additional file 20). Protein names are colored by species (green is *Caenorhabditis elegans*, blue is *C. briggsae*, and red is *C. remanei*). In addition to identifiers, each name includes the genome position of the corresponding gene. Open circles on branches indicate a branch support value of 0.9 or higher, as computed by *phyml-alrt*. The scale bar indicates number of amino acid changes per site. Probable strict ortholog trios are marked with filled black squares. A representative gene expansion in *C. elegans *is marked and a view of the gene arrangement is expanded to the right (adapted from the WormBase genome browser, WS170). An alignment of four of the *C. elegans *proteins from this gene expansion is shown in the lower right. Blue coloring is proportional to amino acid conservation.

For each chemoreceptor family, proteins from all three species were combined and a maximum likelihood tree was built with approximate likelihood ratio test (aLRT) branch supports [35]. Figures representing these trees for each of the 19 families are given in Additional files 14 to 32. A representative section of one of the trees is shown in Figure 1. All of the families were characterized by a substantial number of apparent gene duplications and probably a roughly balanced number of gene losses (birth-death evolution). Additional file 10 gives summary statistics that capture these properties for each family. At one extreme, the *C. elegans *srbc, srw, and srz families have very few clear orthologs across the three species. These trees instead are dominated by species-specific expansions, which presumably arose by a process of ongoing gene duplication and loss on each lineage. At the other extreme, more than half of the genes in the *C. elegans *sre and srxa families have single orthologs in both *C. briggsae *and *C. remanei*, although even these relatively stable families have substantial numbers of apparent gene duplications and losses. To test whether incomplete genome sequence or analysis problems might contribute to these patterns, we applied the same gene prediction and tree analysis to 30 metabotropic GPCRs and to 39 FMRFamide receptor-related genes from *C. elegans*. Nearly all of these genes had single orthologs in *C. briggsae *and *C. remanei*, as summarized in Additional file 10. Similar analysis of other stable gene families gave similar results (data not shown). We conclude that very few genes are missing from the *C. briggsae *and *C. remanei *sequence assemblies and that our methods of gene annotation are of sufficiently high quality to give good estimates of family stability.

**Figure 2:**
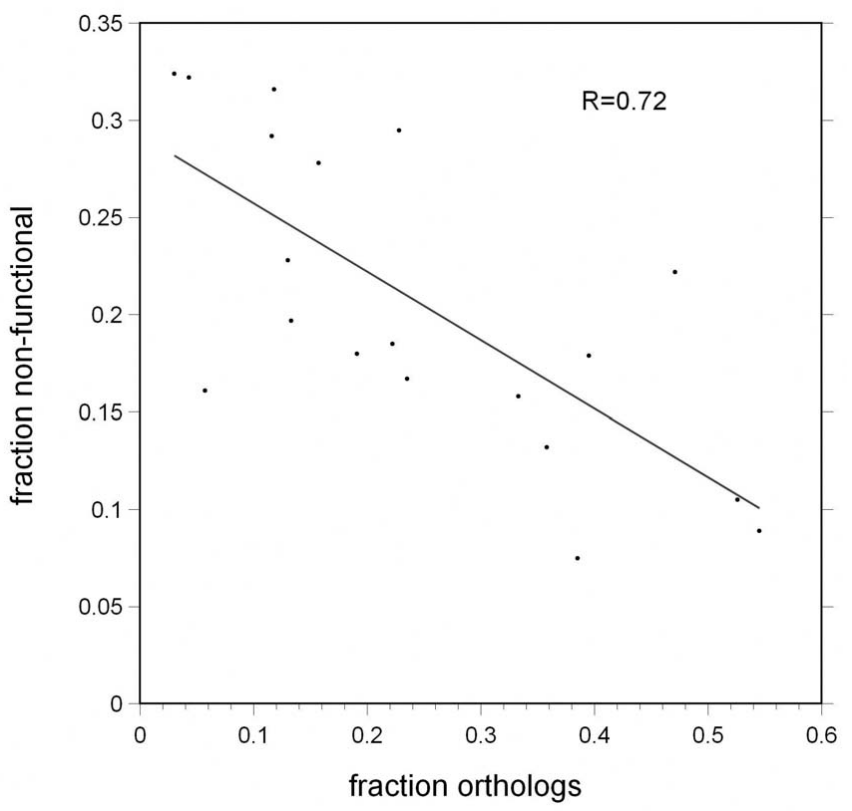
**Negative correlation between orthology and nonfunctional gene frequencies in *Caenorhabditis elegans***. Each of the 19 gene families is plotted once. The X-axis is the fraction of *C. elegans *genes in the family with single orthologs in both *C. briggsae *and *C. remanei*. The Y-axis is the fraction of *C. elegans *genes in the family with probable defective alleles in the N2 reference genome sequence. The correlation shown is much stronger than that between family size and fraction of defective genes (see Additional file 9). *R *is the Pearson correlation coefficient.

The fraction of defective genes in a family in *C. elegans *was well correlated with the family-specific rate of gene duplication and loss, as indirectly inferred from the fraction of orthologs in the protein tree (Figure 2). This correlation fits with the idea that gene duplication is balanced by gene loss. One route to gene loss is the fixation of a defective allele in the population followed by eventual deletion of the now neutrally evolving segment of DNA. Although it is unclear what fraction of defective genes in the sequenced N2 genome are fixed pseudogenes [28], it is likely that many are, since they have more than one obvious defect. These are presumably gene family members on their way to complete gene loss. Assuming pseudogenes in different families are deleted from the genome at a similar rate, the frequency of such pseudogenes is expected to reflect the family-specific rate of gene loss.

### Gene duplication and genome position

Patterns of genome location for all 19 families were analyzed in *C. elegans*, where the genome sequence is complete. The degree of gene clustering and predominant chromosomal location for each family is summarized in Additional file 10 and color-coded positions of genes in all families are shown in Additional file 33. Most genes in each family were located on a single chromosome (or two chromosomes for the sri, sru, and srz families). These patterns are consistent with an ancestral gene on one chromosome and largely local gene duplication events giving rise to the family over time. Even at the superfamily level, each of which presumably arose from an even older ancestral gene, this chromosome bias is apparent: most Str and Srg superfamily genes are located on chromosome V and most Sra superfamily members are located on chromosome II. Detailed inspection of protein trees and gene locations suggests that most exceptions to genes remaining on their ancestral chromosome arose by transposition of single genes followed by further duplication at the new chromosomal home. These results support the conclusion that evolutionarily retained translocation events are extremely rare in *Caenorhabditis *species [36] and that the chromosome is a meaningful unit of long-term evolution in nematodes. Similar inferences are published for the srh, str, and srj families [3,4].

### Lack of evidence for positive selection

Genes in the srz chemoreceptor family are under positive selection in both *C. elegans *and *C. briggsae *[6]. In that work, maximum likelihood d_N_/d_S _tests were conducted on paralog samples from most of the chemosensory receptor families described here. We carried out additional systematic tests among all 19 chemoreceptor families. These analyses confirmed positive selection in the srz family and produced no clear evidence of positive selection in any of the other families (Additional file 34). It is important to note that the d_N_/d_S _method used for these tests is sensitive only to persistent long-term positive selection. Methods for testing more recent selection events are not currently applicable with the limited population sequence data available in *Caenorhabditis*.

### Promoter E-box distribution

One possible selective force for expansion in gene number is an increased need for diversified chemosensory receptors expressed in specific sensory neurons. Little is known about how promoter sequences control cell-type specific expression of chemoreceptor genes in *C. elegans*. However, one promoter sequence, called the E-box, has been shown to be enriched in *srh *and *sri *chemoreceptor genes and to drive expression in the chemosensory neuron ADL [37]. To test the distribution of E-boxes among all chemoreceptor genes, we extracted 20,569 promoter sequences that include 1675 chemoreceptor family loci. To reduce non-specific noise, analysis was restricted to the region from -200 to -20 from the predicted translation start, where E-boxes are concentrated (data not shown). Each promoter was scanned for its best match to an E-box profile and the 500 best-scoring matches were analyzed for their distribution among gene families (Table 1). There was strong enrichment of E-boxes in chemoreceptor genes as a whole, and this was due entirely to high match frequencies in four families. The other 15 chemoreceptor families had an E-box match frequency similar to the genome as a whole; we conclude that few if any genes in these families are likely to be regulated by E-box binding factors. The sre, srh, sri, and srz families were strikingly enriched in E-boxes, with 25% to 50% of their promoters in the high-scoring set. If E-box driven expression in ADL neurons generalizes to the other chemoreceptor families, then ADL likely expresses about 200 chemoreceptors, largely from the sre, srh, sri, and srz families. These four families are very diverse, representing three different superfamilies. These results suggest that the function of ADL sensory neurons requires expression of a large and diverse set of chemoreceptors. Bitter taste sensory neurons in mammals and insects also express multiple receptors [38], an unusual pattern that may be important for broadly tuned aversion to noxious compounds [39,40]. We speculate that the aversion-mediating neuron ADL has a similarly broad sensitivity in nematodes.

**Table 1:** E-box matches for all promoters

Superfamily	Family	Promoters	E-box Matches	Percent with E-box	*P*-value
Sra	sra	39	0	(0)	NS
Sra	srab	27	1	(3.7)	NS
Sra	srb	19	0	(0)	NS
solo	srbc	64	0	(0)	NS
Str	srd	71	0	(0)	NS
Sra	sre	55	14	25.5	<0.0001
Srg	srg	69	4	5.8	NS
Str	srh	304	104	34.2	<0.0001
Str	sri	78	39	50.0	<0.0001
solo	srsx	35	1	(2.9)	NS
Srg	srt	73	1	(1.4)	NS
Srg	sru	45	4	8.9	0.02
Srg	srv	36	2	5.6	NS
solo	srw	148	8	5.4	0.04
Srg	srx	137	3	2.2	NS
Srg	srxa	16	2	12.5	NS
solo	srz	104	51	49.0	<0.0001
Str	str/srj	323	9	2.8	NS
	All SR	1675	246	14.7	
	All genes	20569	500	2.4	NA

E-box hits are the 500 highest scoring motif matches in promoters from -200 to -20 from the translation start codon. Percent values based on zero or one hit are in parentheses to indicate high uncertainty. The uncorrected *P*-value shown was determined by comparison with the number of hits in all promoters (Fisher's exact test for small families and the χ-square approximation for large families). After Bonferoni correction for multiple testing the sru and srw *P*-values are not significant. The str and srj families are closely related and were analyzed together. A few small chemoreceptor families are not shown individually, so numbers shown do not add up to the SR total. SR, all members of putative chemoreceptor families; NA, not applicable; NS, not significant.

### Brief family summaries

#### Sra superfamily

##### sra family

This small published family [7] includes a single large gene expansion in *C. elegans*, all of which are in a cluster on the right arm of chromosome I (Additional file 14). This expansion probably arose following the transposition of a single *sra *gene from chromosome II, where most other members of the family reside. Apart from this part of the family, there is a relatively high frequency of orthology, suggesting that many *sra *gene functions are evolutionarily stable. Most SRA proteins have two potential disulfide bonds that could join extracellular domain 1 with 2 and domain 2 with 3 (Additional file 2). Five SRA proteins lack the first cysteine pair, which suggests this pairing pattern. There are no strongly characteristic sequence motifs conserved in the family. The sra, srab, and srb families are closely related and a few genes are difficult to place in a specific family. The family corresponds to existing Pfam profile PF02117.

##### srab family

This small published family [7] includes a single large gene expansion in *C. elegans*, all but one of which are in a cluster on the left arm of chromosome V (additional file 15). Apart from this part of the family, there is a relatively high frequency of orthology, suggesting that many *srab *gene functions are evolutionarily stable. SRAB proteins have two conserved potential disulfide bonds in the same positions as those in the sra family (Additional file 2). There is a protein segment in and near TM domain 2 that contains several highly conserved H, N, R, and D residues, which is characteristic of the srab family. The family has been assigned to the new Pfam profile PF10292. Also see the sra family notes above.

##### srb family

This small family includes no large *C. elegans *gene expansions, but has one modest expansion in *C. remanei *and occasional duplications and losses throughout the tree (Additional file 16). The overall frequency of orthologs is relatively high, suggesting that many *srb *gene functions are evolutionarily stable. There is one conserved potential disulfide bond that could join extracellular domains 1 and 2 in approximately the same position as one of the potential disulfide bonds in the sra and srab families (Additional file 2). At or near the inner end of TM domain 2 there is a nearly invariant FHxN sequence, which is shared with some SRAB and SRE proteins but is otherwise characteristic of the srb family. The family corresponds to existing Pfam profile PF02175. Also see the sra family notes above.

##### sre family

This medium-sized family includes two modest gene expansions in *C. elegans*, each located in a cluster on the right arm of chromosome II (Additional file 19). In other parts of the tree there is a high frequency of orthology, suggesting that many *sre *gene functions are evolutionarily stable. There are no conserved potential disulfide bonds in the family (Additional file 2). Most SRE proteins share the FHxN sequence with SRB proteins and there is a highly conserved R/KFQxxEN sequence in intracellular loop three that is characteristic of the family. There is marked variation in the length of the second extracellular loop, with about half of SRE proteins having an approximately 20 amino acid insertion relative to the others. This variant is found in all three *Caenorhabditis *species and correlates with other sequence characters in the protein tree. The family corresponds to existing Pfam profile PF03215.

#### Srg superfamily

##### srg family

This medium-sized family includes two modest gene expansions in *C. elegans*, one in a cluster on the left arm of chromosome V and another mostly in a cluster on the left arm of chromosome II (Additional file 20). There are lower rates of gene duplication and gene loss in most of the rest of the tree, giving the family as a whole a moderately high frequency of orthologs. There is a single potential disulfide bond in about half of SRG proteins that could join extracellular domains 1 and 2 (Additional file 3). There is a nearly invariant W residue near the inside end of TM domain 4 that is characteristic of the family. The family corresponds to existing Pfam profile PF02118.

##### srt family

This published family [41] is medium sized and gene duplications and losses are fairly evenly distributed across the protein tree, with several small expansions in *C. elegans*, all located in clusters on the left arm of chromosome V (Additional file 25). There is a relatively low frequency of orthologs. There is one potential disulfide bond in most SRT proteins, which could join extracellular domains 1 and 2 (Additional file 3). There are two nearly invariant W residues, one in TM domain 3 and another in TM domain 7, which are characteristic of the family. The family has been assigned to the new Pfam profile PF10321.

##### sru family

Instability in this medium-sized family is fairly evenly distributed across the protein tree, with several small expansions in *C. elegans*, located mostly in clusters on the left and right arms of chromosome V (Additional file 26). The family is notably larger in *C. remanei*, mostly as a result of two large expansions on the tree. The *C. remanei *assembly is not adequate to fully assess genome clustering of these two gene expansions, but there is clearly some clustering on specific supercontigs. There is a relatively low frequency of orthologs. There is one potential disulfide bond in all but a few SRU proteins that could join extracellular domains 3 and 4 (Additional file 3). There is a highly conserved protein segment in and near the inner end of TM domain 7 that is characteristic of the family. The family corresponds to existing Pfam profile PF02688.

##### srv family

This medium-sized family includes a single small gene expansion in *C. elegans *that is in a cluster near the center of chromosome IV. *C. remanei *has several small expansions scattered across the tree (Additional file 27). The rest of the tree is characterized by modest frequencies of gene duplication and loss, giving an overall frequency of orthology that is relatively low. There are no conserved potential disulfide bonds in the family and no other obvious distinguishing sequence characteristics (Additional file 3). The family has been assigned to the new Pfam profile PF10323.

##### srx family

This large family includes several modest gene expansions in *C. elegans*, mostly in gene clusters on the left and right arms of chromosome V and on the left arm of chromosome II (Additional file 29). Outside of these regions of the tree there are many orthologs, giving the family overall an average frequency of orthology. There is one conserved potential disulfide bond between two residues in extracellular domain 3 (Additional file 3). There is a nearly invariant NR motif in TM domain 3 that is found only in SRX and SRXA proteins. The family has been assigned to the new Pfam profile PF10328.

##### srxa family

This small family includes a relatively small number of gene duplications and losses spread evenly across the tree (Additional file 30), giving the family overall a relatively high frequency of orthologs and suggesting that many *srxa *functions are evolutionarily stable. All SRXA proteins have one potential disulfide bond between two residues in extracellular domain 3 (Additional file 3), a characteristic that is shared only with the related srx family. The family corresponds to existing Pfam profile PF03383. Also see the srx family notes above.

#### Str superfamily

##### srd family

This medium-sized family includes two moderately large gene expansions in *C. elegans *(Additional file 18). One expansion corresponds to a gene cluster on the right arm of chromosome V, but the other includes genes on chromosomes III and V and presumably involves one or more transposition events. Outside of these parts of the tree there are relatively few gene duplications and losses, giving the family as a whole a moderate frequency of orthologs. Most SRD proteins have one potential disulfide bond between two residues in extracellular domain 2, a pattern that is found only in this family (Additional file 4). It shares with the srh and sri families a highly conserved PYR sequence at or near the inner end of transmembrane (TM) domain 7. The family has been assigned to the new Pfam profile PF10317.

##### srh family

This published family [4] is the largest chemoreceptor gene family in *C. elegans*. The tree includes several large to moderately large gene expansions in *C. elegans*, mostly in gene clusters on the left and right arms of chromosome V (Additional file 21). There are no conserved potential disulfide bonds (Additional file 4). There is a conserved HG motif in TM domain 7 that characterizes most of the family. Several other short sequence motifs are shared only with the sri family. The family has been assigned to the new Pfam profile PF10318.

##### sri family

This medium-sized family is closely related to the srh family, and includes several moderately large gene expansions in *C. elegans*, mostly in gene clusters in several places in the genome (Additional file 22). *sri-1 *and *sri-2 *are an outgroup to the rest of the family and in some analyses tree with the srh family. There are no conserved potential disulfide bonds (Additional file 4). There is a conserved CF motif at or near the inner end of TM domain 3 that characterizes most members of the family. The family has been assigned to the new Pfam profile PF10327. Also see srh family notes.

##### srj family

This medium-sized published family [3,5] is closely related to the str family (some older literature refers to the srj family as 'stl'). The family includes one large gene expansion in *C. elegans *mostly in one gene cluster on the left arm of chromosome V (Additional file 23). Most of the rest of the tree includes lower frequencies of gene duplications and losses and the family overall has few orthologs. *srj-1 *is highly divergent from all other *srj *genes but belongs in this family based on rooted trees. There are no conserved potential disulfide bonds (Additional file 4). SRJ proteins can be distinguished from STR proteins based on a nearly invariant RC motif near the center of TM domain 3. The family has been assigned to the new Pfam profile PF10319.

##### str family

This published family [3,5] is the second largest chemoreceptor gene family in *C. elegans*. There are several moderately large gene expansions in *C. elegans*, mostly in gene clusters on the right arm of chromosome V, and several gene expansions in *C. briggsae *and *C. remanei *(Additional file 32). Most of the rest of the tree is also characterized by a substantial frequency of gene duplications and losses, and overall the family has few orthologs. This family includes ODR-10, the only chemoreceptor with a known specific function [9-11]. ODR-10 is on a relatively stable part of the protein tree and has orthologs in *C. briggsae *and *C. remanei*. There are no conserved potential disulfide bonds but there are several nearly invariant residues in and near TM domain 6 that are shared only with the srj family (additional file 4). The family has been assigned to the new Pfam profile PF10326.

#### Other families

##### srbc family

This medium-sized family is dominated by three large gene expansions in *C. elegans*, mostly in gene clusters in several places on chromosome V (Additional file 17). There are also gene expansions in *C. briggsae *and *C. remanei *but they involve fewer genes, so that the srbc family is much larger in *C. elegans*. There are very few orthologs in the family. There are two invariant potential disulfide bonds, both among residues in extracellular domain 3, a pattern that is characteristic of the family (Additional file 5). The spacing between these cysteine residues is nearly invariant but there is substantial variation in the specific sequences. It seems likely that the disulfides form a framework for a ligand-binding domain unique to this family. The family has been assigned to the new Pfam profile PF10316.

##### srsx family

This small family includes a few recent gene duplications in *C. elegans*, mostly in local pairs on the right arm of chromosome V (Additional file 30). Most of the tree has very little gene duplication and loss, giving the family overall a relatively high frequency of orthologs and suggesting that many *srsx *functions are evolutionarily stable. There is one invariant potential disulfide bond that could join extracellular domains 2 and 3 (Additional file 6). About 15% of SRSX proteins have an approximately 12 amino acid insertion in intracellular domain 3 that is highly conserved, suggesting a distinct G-protein interaction for this group. There is a nearly invariant GN sequence near the middle of TM domain 1 that is characteristic of the family. The family has been assigned to the new Pfam profile PF10320.

##### srw family

This large family is the only one with a clear sequence relationship to other known GPCR proteins: it is weakly but clearly related to several groups of neuropeptide receptors, including the myosuppressin receptor family in insects and the FMRFamide receptor family in nematodes (data not shown). Despite this relationship, it is unlikely that SRW proteins function as neuropeptide receptors because they are subject to a high rate of gene duplication and loss, in stark contrast to members of the known peptide receptor families (Additional file 10 and data not shown). We speculate that SRW proteins function as receptors for environmental peptides. The family includes several moderate to large gene expansions in *C. elegans *and similar, albeit smaller, expansions in *C. briggsae *and *C. remanei *(additional file 28). The expansions in *C. elegans *correspond to several gene clusters mostly on the left and right arms of chromosome V. There is one highly conserved potential disulfide bond that could link extracellular domains 2 and 3 (Additional file 7). There are two nearly invariant H residues that are characteristic of the family, one in TM domain 1 and the other in TM domain 7. The family corresponds to existing Pfam profile PF06976.

##### srz family

This moderately large published family [6] is characterized by a high rate of gene duplication and gene loss and is uniquely subject to strong positive selection on the extracellular face of the protein. The tree is dominated by gene expansions specific to each species and indicates very few orthologs (Additional file 31). There are no conserved potential disulfide bonds and the proteins have diverse length and sequence in all four extracellular domains (Additional file 8). There are several conserved sequence motifs in or very near TM domains that are characteristic of the family. The family has been assigned to the new Pfam profile PF10325.

## Discussion

### Gene number and diversity

The chemoreceptor genes in *Caenorhabditis *are strikingly abundant and diverse. In contrast, mammalian olfactory receptor genes are abundant but have relatively limited diversity, with nearly all of the genes belonging to a single gene family [16]. For example, among putative functional olfactory receptors in human, dog, and mouse, the average pairwise amino acid identity is about 0.37 (0.369 among 377 *Homo sapiens *proteins, 0.370 among 473 *Canis familiaris *proteins, 0.384 among 1144 *Mus musculus *proteins, National Center for Biotechnology Information (NCBI) RefSeq24, data not shown). This level of diversity is lower than within any single *C. elegans *family (Additional file 9). Fish and amphibians have more diverse olfactory receptor genes, yet even in these groups all known genes fall into a single superfamily, with significant sequence relatedness between any given pair of olfactory receptor proteins [16]. Insects have much smaller chemoreceptor gene families [25,27,42]. *Caenorhabditis *species have seven superfamilies of putative chemosensory receptor genes, with no significant sequence similarity across superfamilies. Why do nematodes have such high diversity in their chemoreceptors? We suggest that this is a result of sensory system emphasis. Unlike vertebrates and insects, nematodes lack vision and hearing. We speculate that chemosensory diversity is thus more central to nematode sensory capabilities, leading to a high genetic investment and high receptor diversity. A similar explanation for the smaller olfactory gene number in great apes relative to rodents has been proposed, based on an increased emphasis on a sophisticated visual system [20].

### The gray pawn hypothesis

Totaled across all 19 families, the putative functional chemoreceptor genes account for about 7% of all *C. elegans *genes. Despite this large genetic investment in a single class of genes, our comparison among the three sequenced *Caenorhabditis *species and gene knockdown studies [43] suggest that few if any of the specific genes are essential for species viability, although subsets are implicated in specific physiological processes such as maintenance of fat content [44]. Instead, individual genes are lost at a high rate and this loss is roughly compensated by duplications among the remaining genes, similar to the olfactory receptor family in mammals [16]. One possible explanation of these patterns is that new duplicate genes diverge and confer some specific selective advantage (positive selection). However, in both nematodes and mammals there is evidence for such positive selection in only small and specific subsets of chemoreceptor genes [6,20,45,46]. Although further analysis might reveal subtler signs of positive selection, it is unlikely to be a major evolutionary force in these families, in contrast to several other gene families in which similar d_N_/d_S _analysis reveals extensive evidence of positive selection (see, for example, [47-49]). What else could account for these patterns? We propose that the function of most of these genes is not to be found in their individual contributions to fitness, but rather in their aggregate function in covering a large phenotype space. In this model, each gene is a nearly insignificant and faceless pawn, but in aggregate these gray pawns form an effective army. Neutral processes may dominate many aspects of evolution in these families, but this alone cannot account for the retention of roughly similar numbers of genes in three highly divergent species. The gray pawn hypothesis suggests that if the number of functional genes in a family drops too much, selective pressure increases to gain new genes by duplication and divergence, and gene number increases by selective retention of new duplicates. If the number of genes increases too much, selective pressure to maintain individual genes decreases, and genes are lost by mutation and drift. The number and diversity of functional genes results from this dynamic equilibrium combined with the species-specific importance of the process mediated by the gene family. Evolution of the immunoglobulin gene families in mammals may also be explained in part by the gray pawn hypothesis.

## Conclusion

We have completed the annotation and description of all 19 large GPCR gene families in *C. elegans *and its relatives *C. briggsae *and *C. remanei*. All of the families are characterized by a substantial number of nonfunctional genes in each species. The rates of gene duplication and gene loss, inferred from protein trees in each family, are substantial in all the families. Despite these dynamics, evidence for positive selection is found in only one of the families. We propose the gray pawn hypothesis to explain these patterns of molecular evolution: most individual genes are of little significance, but as a group they are required to cover a broad ligand space. This is achieved by maintaining a large and diverse repertoire of chemoreceptors in which new genes arise by duplication and divergence and others are lost by mutation and genetic drift.

## Methods

### Family identification

The *C. elegans *genome contains a large number of GPCR genes; other than sharing a seven TM domain (7-TM) structure there is no universal sequence signature that can be used to identify all of the genes. A variety of approaches were taken to ensure that all or nearly all GPCR genes were identified. First, multiple *blastp *and *psi-blast *searches [50] were conducted using as queries previously identified *C. elegans *GPCR proteins and those from other metazoans. Second, since most families of GPCR genes in *C. elegans *are found in genome clusters, genes adjacent to identified GPCR genes were manually tested for a 7-TM domain structure or relationship to previously identified GPCR proteins by *blastp *searches against the 'nr' dataset at NCBI. Third, the entire predicted protein set for *C. elegans *was analyzed by TM hidden Markov model (HMM) [51], and all proteins predicted to have five to eight TM domains (to allow for some TM and gene prediction inaccuracy) were tested by manual inspection and by *blastp *searches against the 'nr' dataset at NCBI. Finally, *blastp *and *psi-blast *searches of the entire predicted *C. elegans *protein set were conducted with all GPCR proteins collected from the previous searches. We think we can be confident that all large GPCR families were identified by these approaches, but a few unique genes or genes in small families might remain unidentified. Nearly all of the genes identified in a recent HMM survey tailored to GPCRs (GPCR HMM) [52] are members of the families described here or of other small families that we had already identified (data not shown). After additional gene model correction and identification of related unpredicted genes (see gene annotation below), these methods identified 1990 genes and pseudogenes that probably encode GPCR proteins. Of these, 111 are members of known families of metabotropic GPCRs, including serotonin, dopamine, acetylcholine, glutamate, and neuropeptide receptor families. Of the remaining genes, 180 were singletons or were in small gene families (10 or fewer genes) that were only weakly related to anything else; these were not analyzed further. The remaining 1699 genes and pseudogenes fell into 19 gene families, six of which are previously described [3-7,41].

### Gene annotation in *C. elegans*

We carried out a largely manual annotation of the entire set of putative chemoreceptors in *C. elegans *before refining gene predictions in *C. briggsae *and *C. remanei*. Within each gene family, all candidate genes were identified by *blastp *searches of the latest WormBase protein predictions (the exact prediction version changed over the several years of this effort). A multiple alignment of these predictions readily identified exceptions to the predominant protein structure, largely a result of fusions to adjacent genes or missing exons. Gene fusions were resolved by splitting at the appropriate intron and extending each half gene to the appropriate Met and stop codon. Missing exons were usually found by using the most similar well-aligned full-length protein as the query in a *tblastn *search of genome sequence. Early in this effort some missing exons were found by hand inspection of genome sequence. Less commonly, specific exons were extended or shortened from the results of similar analysis. In some cases, these corrections required the incorporation of an exon containing an internal stop codon or frameshift; in these cases the gene was judged to be defective. In a few other cases, exons that were clearly required to complete a full-length protein sequence could not be joined with good quality splice junctions; these genes were also judged to be defective. Usually these manually curated changes were very obvious improvements to existing predictions but occasionally some subjective judgment was applied. All corrected predictions were communicated to WormBase annotators and nearly all were accepted as appropriate. Rarely, WormBase annotators identified an anomaly or provided an alternative prediction; these cases were resolved by discussion. Although every effort was made to obtain correct gene models, there is little doubt that occasional improvements will continue to be made as a result of experimental analysis. The set of genes analyzed here is consistent with the current WormBase at the time of writing (WS170) [53].

### Gene annotation in *C. briggsae* and *C. remanei*

Gene prediction sets in these two species were insufficiently accurate for our needs. We obtained improved protein sets for each family as follows, using the briggsae_cb3 (*C. briggsae*) and the PCapV2 (*C. remanei*) genome assemblies [32]. First, all full-length *C. elegans *proteins from the family were used as queries in *blastp *searches of the brigpep (*C. briggsae*) and wum (*C. remanei*, Wash U merged) protein predictions. The collected family members from all three species were aligned and proteins from *C. briggsae *and *C. remanei *with missing, inserted, or fused regions were discarded. This produced a set of proteins across all three species that were likely to be full length and correctly predicted (called setA). As expected because of our intensive annotation of the *C. elegans *genes, there were more setA proteins from that species, but many proteins from the other two species were also found. These proteins were used as guides in a *GeneWise *prediction pipeline as described elsewhere [47]. For *C. briggsae*, this procedure produced two prediction sets: one from brigpep and one from *GeneWise*. These sets were combined and proteins that were less than half length (compared with the mean length of setA) were discarded. Predictions from brigpep that spanned two or more *GeneWise *predictions were discarded (these resulted from inappropriate fusion or hybrid gene prediction, data not shown). The remaining proteins were used as queries in a *blastp *search of setA. For proteins whose gene models overlapped in the genome, one best protein was chosen (based on its best *blastp *score to setA) with a heuristic bias toward proteins of approximately the mean length for the family. When scores were equal (for example, identical predictions) the brigpep gene name and protein were retained. Summing over all of the chemoreceptor families, about one-third of the best protein predictions were from brigpep and the rest were from *GeneWise*. Inspection of trees and alignments indicated that the combined protein set was clearly superior to either brigpep or *GeneWise *alone (data not shown). For *C. remanei*, the same procedure was adopted, except that both the wum and genefinder prediction sets were combined with the *GeneWise *set and the best protein among the three was chosen. When scores were equal, the wum gene was preferred, followed by the genefinder gene, and lastly the *GeneWise *gene. About one-quarter of the best protein predictions were from wum, about one-tenth were from genefinder, and the rest were from *GeneWise*. The high rate of improved gene predictions with *GeneWise *in both *C. briggsae *and *C. remanei *is presumably the result of large numbers of setA proteins available to guide high-quality predictions of other genes.

For comparison, we ran the same prediction pipeline starting with sets of 30 metabotropic GPCR proteins and 39 FMRFamide receptor-related proteins from *C. elegans*. In both cases, this resulted in numbers of gene predictions in *C. briggsae *and *C. remanei *that were nearly identical to *C. elegans *(Additional file 10). When tree analysis was applied to these predictions, at least 90% of the genes from the three species were clear 1-1-1 orthologs. The few exceptions could result from genome sequence or assembly problems, but it is plausible that they instead represent rare gene losses and duplications. These results indicate that the assembly and sequence coverage and quality in *C. briggsae *and *C. remanei *are excellent, and they suggest that our prediction pipeline is accurate.

### GPCR membership

To critically assess whether the families studied here are truly GPCR proteins, a representative protein from each questionable family was used to initiate a *psi-blast *search (default parameters) on the NCBI 'nr' data set (July, 2007) [54]. For each family, among the many robustly recruited GPCRs of known function were: sra, melanin-concentrating hormone receptor (mouse MCHR-1); srab, thyroid-stimulating hormone receptor (human TSHR); srb, melanin-concentrating hormone receptor (mouse MCHR-1); srbc, angiotensin II receptor type 1 (human MAS1); sre, sphingolipid G-protein coupled receptor 1 (human EDG1); srg, opsins (many species); srsx, somatostatin receptor (mouse Sstr2); srt, opsins (many species); sru, opsins (many species); srv, opsins (many species); srx, melatonin receptor 1A (human MTNR1A); and srxa, melanin-concentrating hormone receptor (mouse MCHR-1). In addition, most of the queries matched one or more Pfam profiles of 7-TM receptors. The srh, sri, str, srj, and srd families are clearly related to each other, and one member of the str family (ODR-10) is a known G-protein coupled odorant receptor in *C. elegans *[9-11]. The srw family is clearly related to various known neuropeptide GPCRs. The srz family gave no *psi-blast *hits outside of its own family in *Caenorhabditis *but gave a weak match to a PFAM 7-pass receptor profile (PF01748) and many members were identified as GPCR proteins by GPCRHMM [52].

### Gene counts and nonfunctional genes

The functional status of genes in chemoreceptor families was very difficult to define accurately, especially in *C. briggsae *and *C. remanei*. Problems included low transcript abundance (resulting in low rates of expressed sequence tag (EST) confirmation), relatively high levels of amino acid divergence even among putative functional genes, incomplete reference sequence (in *C. briggsae *and *C. remanei*), and uncertainty in the reference sequence (especially in *C. briggsae *and *C. remanei *for stop codon and frameshift defects, where a single nucleotide error could misclassify a gene). In addition to these difficulties, it has been shown that several srh and str family members with defective alleles in the reference *C. elegans *N2 sequence have likely functional alleles in other wild isolates [28]. In Additional file 9 we list the number of probable functional and defective genes in the N2 genome of *C. elegans*. In Additional file 10, we list gene counts based on the following heuristic criteria. First, all best predicted family proteins that were less than half the length of the family average were discarded as probable gene fragments [3-5]. Second, all of the remaining proteins were multiply aligned and alignment columns with 30% or more gaps were removed, which served to remove regions of weak alignment and to remove terminal and internal insertions in *C. briggsae *and *C. remanei *predictions, which are common artifacts of gene prediction. Finally, in this degapped alignment, proteins that included 70% or more of the remaining alignment were retained and counted as genes.

### Protein trees

For each family, all of the best protein predictions that were at least half the length of the family average were collected from the three species and a first-pass *clustalw *alignment was made (default parameters) [55]. Alignment positions with 30% or more gaps were removed, and proteins encoding less than 70% of the remaining alignment were discarded. A few additional proteins were removed in some families based on very long branch lengths in a preliminary maximum likelihood tree (these appeared to be associated with mispredictions or long, poorly alignable regions). The culled protein sets (given in Additional files 11 to 13) were used to construct final trees and make gene counts for Additional file 10. The culled proteins were aligned again with *clustalw *and positions with 30% or more gaps were removed to reduce the influence of poorly aligned regions on the tree. A maximum likelihood tree was computed using *phyml-alrt *(JTT matrix, six rate categories, gamma parameter 1.0), which also computes aLRT branch support in computing time compatible with very large trees [35]. For families clearly related to other chemoreceptor families, the tree was rooted by inclusion of a sampling of proteins from the closest related family. The srbc, srsx, srw, and srz families were left unrooted.

### Family and superfamily classification

Genes were classified as being in the same superfamily when any significant sequence similarity was detected among them using pairwise *blastp *searches. Within superfamilies, genes were classified into families somewhat arbitrarily, in part according to historical family assignments and in part by eyeball clustering in trees of the *C. elegans *proteins (Additional files 35 to 37). Mean pairwise sequence identities within each family are between 0.2 and 0.3 (Additional file 9), so the families are roughly comparable in sequence diversity.

### Pairwise identity measures

All proteins for comparison were multiply aligned using *clustalw*, and alignment columns with more than 30% gaps were removed to reduce the effects of poorly aligned regions. From the resulting multiple alignment, the fraction amino acid identity between all pairs of proteins was determined and averaged. The gap removal step is sensible but had relatively little effect; for example, without gap removal the srh family had 0.226 mean pairwise identity, compared with 0.235 with gap removal.

### Transmembrane domains

Approximate locations of TM domains in each family were assessed by a combination of hydropathy plots of multiple alignments, analysis of a number of single family members by TMHMM [51], and hand reconciling of different family predictions for related families.

### Analysis of positive selection

Tests for positive selection were based on maximum likelihood analysis of d_N_/d_S _values among *C. elegans *paralogs, essentially as described previously [6]. Briefly, well-aligned full-length genes for each family were analyzed. A family protein tree was used to identify all clades of closely related genes that had appropriate total tree lengths for effective maximum likelihood analysis [56]. The protein alignment for each such clade was visually inspected and individual sequences with regions of questionable alignment were removed. A codon alignment and maximum likelihood protein tree for each clade were analyzed by *codeml *models 7 and 8 (three starting omega values, gaps removed). Key results from this analysis are reported in Additional file 34. A total of 135 clades were analyzed from all families combined, and significant positive selection was found only in the srz family (see also [6]).

## Abbreviations

aLRT: approximate likelihood ratio test; d_N_: the number of nonsynonymous codon changes per nonsynonymous site; d_S_: the number of synonymous codon changes per synonymous site; EST: expressed sequence tag; GPCR: G protein coupled receptor; HMM: hidden Markov model; My: million years; NCBI: National Center for Biotechnology Information; TM: transmembrane domain.

### Notation

Gene names are all lower case italicized (for example, *sri-1*).

Protein names are all upper case not italicized (for example, SRI-1).

Family names are all lower case not italicized (for example, sri).

Superfamily names have first letter upper case not italicized (for example, Str).

## Authors' contributions

JHT and HMR collaborated in all aspects of the study. All authors read and approved of the final manuscript.

## Supplementary Material

Additional file 1 **All predicted *Caenorhabditis elegans *chemoreceptor proteins, including those encoded by putative defective genes.** The '~' character in last field of the fasta name indicates that the protein is likely to be defective, followed by a code for the nature of the probable defect: '#' indicates a deletion, '*' indicates a stop codon, and '!' indicates some other defect (usually a splice-site defect). Each putative defect is listed separately, so names with more than one defect code have multiple defects and are likely to represent fixed pseudogenes. We have worked closely with WormBase in updating gene models; other than defective genes, the vast majority of these predictions should correspond exactly to the current WormBase model (WS170 frozen release). Click here for file

Additional file 2 Alignments of Sra superfamily proteins. Each panel shows 20 randomly sampled full-length members of one family within the Sra superfamily. Background shading is proportional to the sum-of-pairs alignment score of each residue relative to the aligned column. Approximate positions of predicted transmembrane domains are marked with bars below the alignment. Domains predicted to be extracellular are marked 'OUT' below the alignment. Probable extracellular disulfide bonds are marked above the alignments. In cases where there is more than one potential disulfide bond, the pairs were inferred by covariance in presence among family members (including other proteins not shown). Click here for file

Additional file 3 **Alignments of Srg superfamily proteins.** See Additional file 2 for the legend. Click here for file

Additional file 4 **Alignments of Str superfamily proteins.** See Additional file 2 for the legend. Click here for file

Additional file 5 **Alignments of srbc family proteins.** See Additional file 2 for the legend. Click here for file

Additional file 6 **Alignments of srsx family proteins.** See additional file 2 legend. Click here for file

Additional file 7 **Alignments of srw family proteins.** See Additional file 2 for the legend. Click here for file

Additional file 8 **Alignments of srz family proteins.** See Additional file 2 for the legend. Click here for file

Additional file 9 **Table presenting the summary of chemoreceptor gene families in *Caenorhabditis elegans*.** Good genes refers to the number of genes we predict will encode functional receptors in the reference N2 genome. Defective genes are all other genes that encode at least half of the family-typical protein; they are about equally divided between those with a single defect (flatliners, potentially defective alleles in N2 [28]) and those with multiple defects (presumed fixed pseudogenes). Click here for file

Additional file 10 **Table presenting the summary of evolutionary properties of chemoreceptor genes from *Caenorhabditis elegans*, *C. briggsae*, and *C. remanei*.** Metabotropic neurotransmitter and FRMF-amide receptor related genes are also shown for comparison. Fraction strict orthologs is the fraction of *C. elegans *genes with single orthologs in both *C. briggsae *and *C. remanei*, as determined the protein tree. Fraction clustered in the genome is the fraction of *C. elegans *genes that have another family member located within five genes in the genome. Tree gene number indicates the number of genes used for protein tree analysis (see Methods for specifics). For the *C. elegans *tree gene number column, the number in parentheses is the number of genes predicted to encode functional receptors in the reference N2 genome. Naively, we expect that a similar fraction of genes from the other two species will be functional in their respective reference genomes. For example, in the srh family there will be (218/294) × 214 functional genes in *C. remanei *and (218/294) × 165 functional genes in *C. briggsae*. Click here for file

Additional file 14 **Maximum-likelihood tree of SRA proteins.***Caenorhabditis elegans *names are green, *C. briggsae *names are blue, and *C. remanei *names are red. Species-specific clades are emphasized by having their branch lines match the species color. The smaller inset is the same tree with names removed, which shows the tree structure more clearly. Open circles on branches indicate a branch support value of 0.9 or higher, as computed by *phyml-alrt*. Strict ortholog trios (1-1-1) are marked with a filled black square. The tree was rooted by inclusion of a sampling of SRAB proteins (not shown). The scale bar indicates number of amino acid changes per site in the large tree. Each name includes a species identifier, gene identifiers, and genome start and end coordinates for the corresponding gene model. The *C. elegans *gene names include both a standard genome project name (for example, F28C12.3) and a genetic gene name (for example, *sra-19*). The *C. briggsae *gene name is the brigpep WormBase name when applicable (for example, CBG04324) or an arbitrarily numbered *GeneWise *prediction number (for example, gw15). The *C. remanei *names are either the WormBase wum gene prediction (for example, wum.4.1), the WormBase genefinder prediction (for example, gf170), or an arbitrarily numbered *GeneWise *prediction number (for example, gw16). The wum or gf names combined with the supercontig number uniquely identify the prediction in the current *C. remanei *prediction set on WormBase. The sequences analyzed are given in Additional files 11 to 13. All trees are available in Newick format upon request. Click here for file

Additional file 15 **Maximum-likelihood tree of SRAB proteins.** See Additional file 14 for the legend. The tree was rooted by inclusion of a sampling of SRA proteins (not shown). Click here for file

Additional file 16 **Maximum-likelihood tree of SRB proteins.** See Additional file 14 for the legend. The tree was rooted by inclusion of a sampling of SRAB proteins (not shown). Click here for file

Additional file 17 **Maximum-likelihood tree of SRBC proteins.** See Additional file 14 for the legend. The tree is unrooted. Click here for file

Additional file 18 **Maximum-likelihood tree of SRD proteins.** See Additional file 14 for the legend. The tree was rooted by inclusion of a sampling of STR proteins (not shown). Click here for file

Additional file 19 **Maximum-likelihood tree of SRE proteins.** See Additional file 14 for the legend. The tree was rooted by inclusion of a sampling of SRA proteins (not shown). Click here for file

Additional file 20 **Maximum-likelihood tree of SRG proteins.** See Additional file 14 for the legend. The tree was rooted by inclusion of a sampling of SRU proteins (not shown). Click here for file

Additional file 21 Maximum-likelihood tree of SRH proteins. See Additional file 14 for the legend. The tree was rooted by inclusion of a sampling of SRI proteins (not shown). Click here for file

Additional file 22 **Maximum-likelihood tree of SRI proteins.** See Additional file 14 for the legend. The tree was rooted by inclusion of a sampling of SRH proteins (not shown). Click here for file

Additional file 23 **Maximum-likelihood tree of SRJ proteins.** See Additional file 14 for the legend. The tree was rooted by inclusion of a sampling of STR proteins (not shown). Click here for file

Additional file 24 **Maximum-likelihood tree of SRSX proteins.** See Additional file 14 for the legend. The tree is unrooted. Click here for file

Additional file 25 **Maximum-likelihood tree of SRT proteins.** See Additional file 14 for the legend. The tree was rooted by inclusion of a sampling of SRX proteins (not shown). Click here for file

Additional file 26 **Maximum-likelihood tree of SRU proteins.** See Additional file 14 for the legend. The tree was rooted by inclusion of a sampling of SRV proteins (not shown). Click here for file

Additional file 27 **Maximum-likelihood tree of SRV proteins.** See Additional file 14 for the legend. The tree was rooted by inclusion of a sampling of SRU proteins (not shown). Click here for file

Additional file 28 **Maximum-likelihood tree of SRW proteins.** See Additional file 14 for the legend. The tree is unrooted. Click here for file

Additional file 29 **Maximum-likelihood tree of SRX proteins**. See Additional file 14 for the legend. The tree was rooted by inclusion of a sampling of SRT proteins (not shown). Click here for file

Additional file 30 **Maximum-likelihood tree of SRXA proteins.** See Additional file 14 for the legend. The tree was rooted by inclusion of a sampling of SRV proteins (not shown). Click here for file

Additional file 31 **Maximum-likelihood tree of SRZ proteins.** See Additional file 14 for the legend. The tree is unrooted. Click here for file

Additional file 32 **Maximum-likelihood tree of STR proteins.** See Additional file 14 for the legend. The tree was rooted by inclusion of a sampling of SRJ proteins (not shown). Click here for file

Additional file 33 **Color-coded positions of all *Caenorhabditis elegans *genes from 19 chemoreceptor families.** The center position of every gene is shown as a grey circle and the chemoreceptor genes are filled and colored. The vertical position of a gene has no significance: it is used merely to space the genes for presentation. Sequence coordinates are shown at the top of each chromosome. Sra superfamily members are shown in shades of green, Srg superfamily members are shown in shades of blue, Str superfamily members are shown in shades of red, and each solo family is shown in a distinct color. Notable concentrations of chemoreceptor genes are apparent on both arms of chromosome II, on the left arm of chromosome IV, and on much of chromosome V, especially both arms. Click here for file

Additional file 34 **Table of the summary of systematic analysis of positive selection.** Analysis included all usable clades of *Caenorhabditis elegans *paralogs from all gene families (see Methods). The specific family sequence file is given for internal reference. The number of sequences in each clade and their mean length in codons is given. Key results from *codeml *analysis are shown for each clade, including the value of the added 11th d_N_/d_S _class from model 8 and the delta maximum-likelihood value used for statistical testing. The Bonferoni corrected *P*-value was computed using a χ-square test with two degrees of freedom. Highly significant evidence of positive selection was found for two clades of srz genes (a more detailed analysis of this family is published elsewhere [6]). Among other families, only one clade of str genes had a marginal significance (set N). Click here for file

Additional file 11 **All *Caenorhabditis elegans *proteins used in protein tree analysis.** The genome start and end position and family are given as part of the fasta name. Coding strand is implied by the order of the two genome coordinates. The list includes some possibly defective proteins if they met our criteria for inclusion in tree analysis (see Methods). Click here for file

Additional file 12 **All *Caenorhabditis briggsae *proteins used in protein tree analysis.** The second field indicates the WormBase gene identifier or an arbitrary *GeneWise *number (see Methods). See also Additional file 11. Click here for file

Additional file 13 **All *Caenorhabditis remanei *proteins used in protein tree analysis.** The second field indicates the wum gene identifier, the genefinder identifier, or an arbitrary *GeneWise *number (see Methods). See also Additional file 11. Click here for file

Additional file 35 **Maximum-likelihood tree of Sra superfamily proteins.** Family members are shown in the same color. Open circles on branches indicate a branch support value of 0.9 or higher, as computed by *phyml-alrt*. Click here for file

Additional file 36 **Maximum-likelihood tree of Srg superfamily proteins.** See Additional file 35 for the legend. Click here for file

Additional file 37 **Maximum-likelihood tree of Srt superfamily proteins.** See Additional file 35 for the legend. Click here for file
