# Supplementary figures and images for: The Caenorhabditis chemoreceptor gene families
Source: BMC Biol. 2008 Oct 6;6:42. doi: 10.1186/1741-7007-6-42 (PMC2576165; doi:10.1186/1741-7007-6-42)

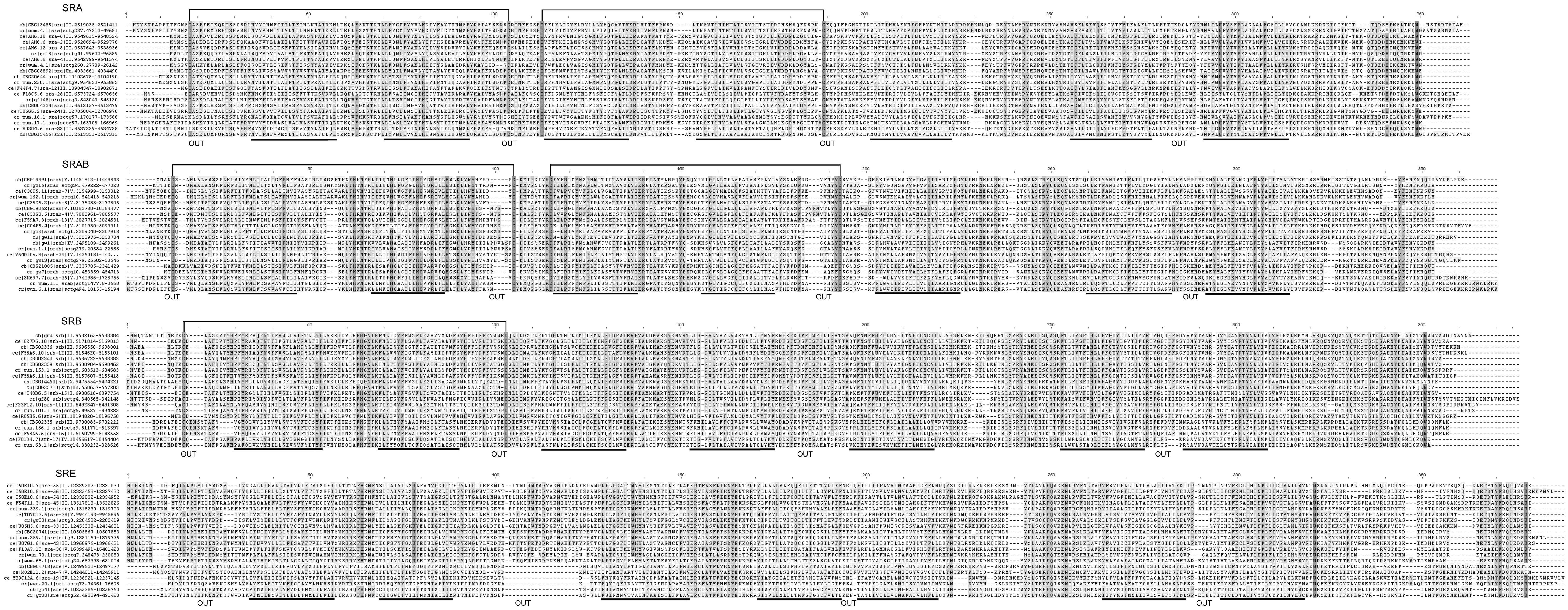

Supplement: Additional file 3 — Alignments of Srg superfamily proteins. See Additional file 2 for the legend. [file 1741-7007-6-42-S3.jpeg]

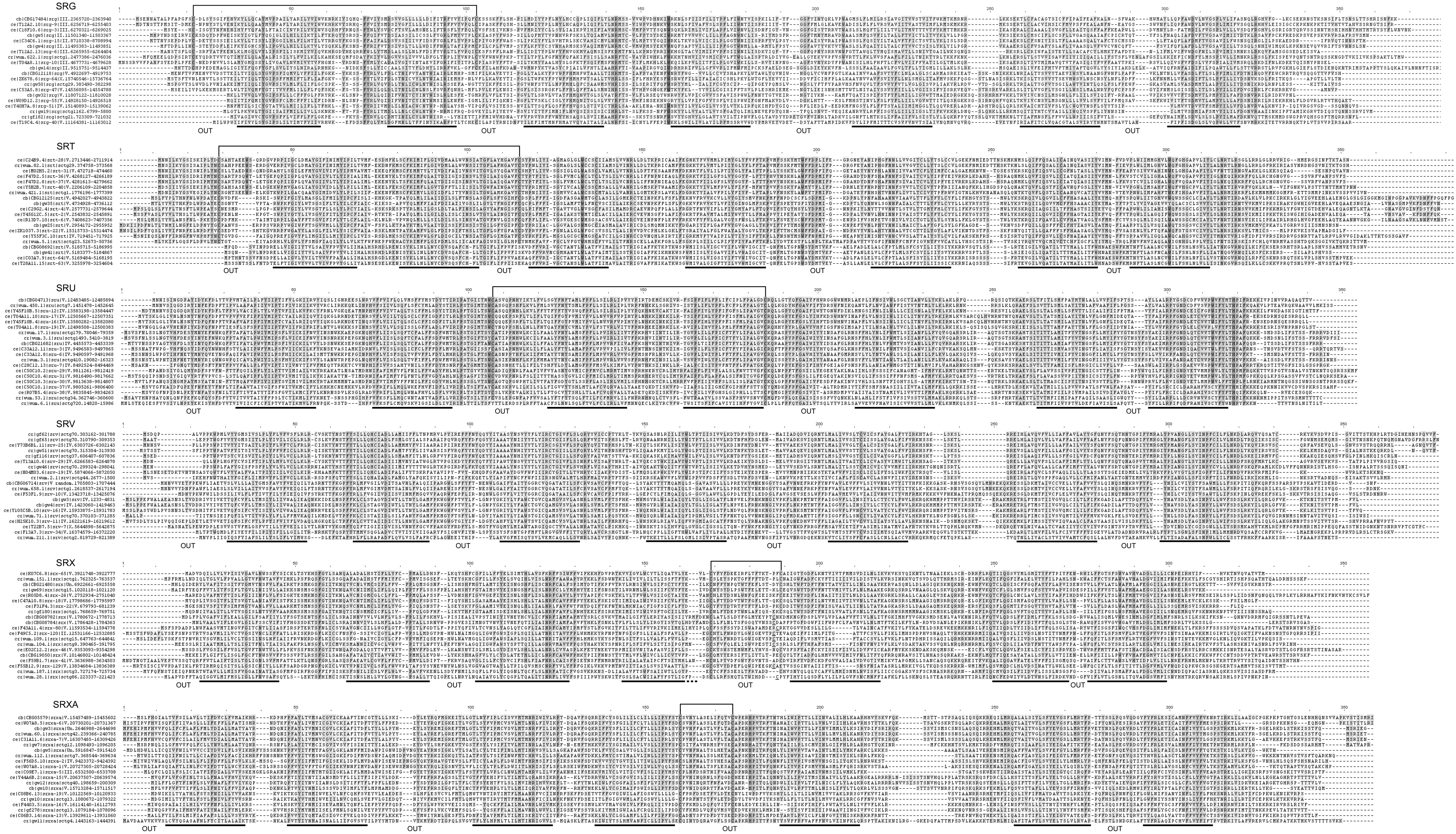

Supplement: Additional file 4 — Alignments of Str superfamily proteins. See Additional file 2 for the legend. [file 1741-7007-6-42-S4.jpeg]

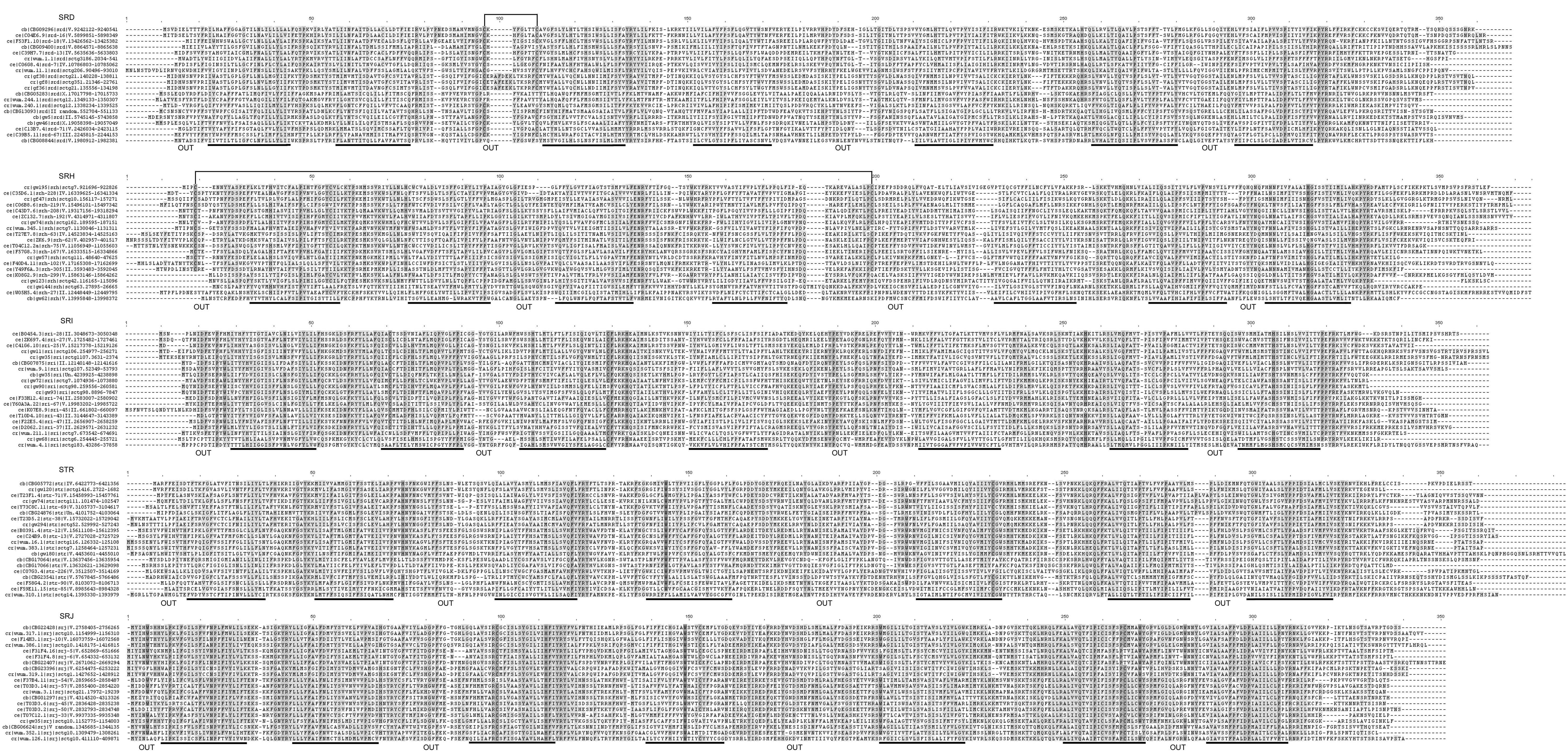

Supplement: Additional file 5 — Alignments of srbc family proteins. See Additional file 2 for the legend. [file 1741-7007-6-42-S5.jpeg]

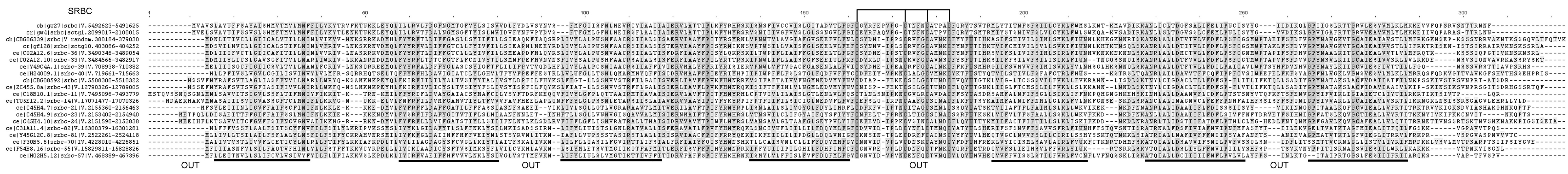

Supplement: Additional file 6 — Alignments of srsx family proteins. See additional file 2 legend. [file 1741-7007-6-42-S6.jpeg]

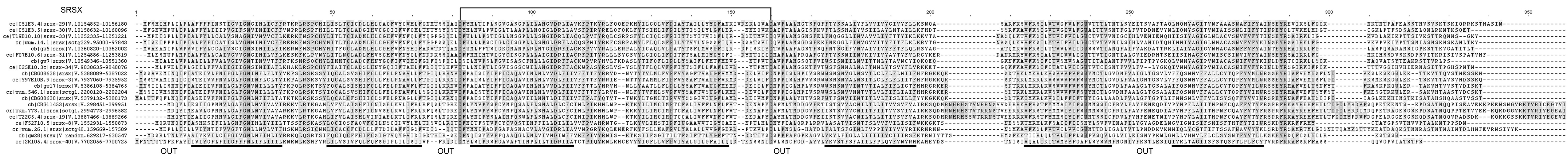

Supplement: Additional file 7 — Alignments of srw family proteins. See Additional file 2 for the legend. [file 1741-7007-6-42-S7.jpeg]

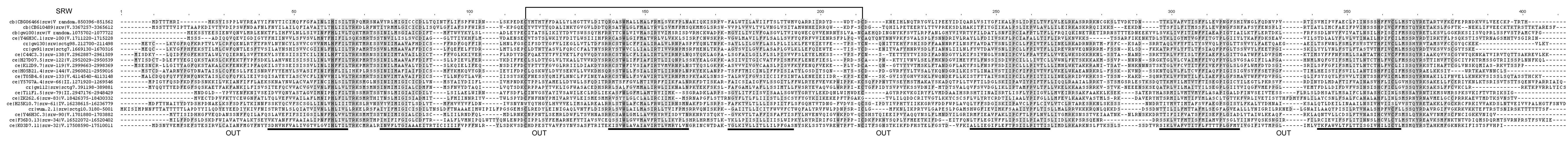

Supplement: Additional file 8 — Alignments of srz family proteins. See Additional file 2 for the legend. [file 1741-7007-6-42-S8.jpeg]

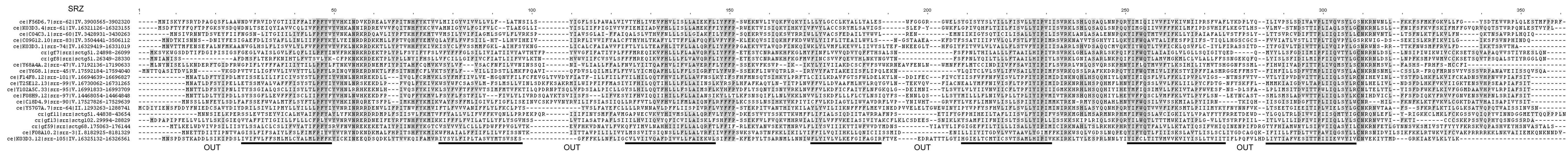

Supplement: Additional file 9 — Table presenting the summary of chemoreceptor gene families in Caenorhabditis elegans. Good genes refers to the number of genes we predict will encode functional receptors in the reference N2 genome. Defective genes are all other genes that encode at least half of the family-typical protein; they are about equally divided between those with a single defect (flatliners, potentially defective alleles in N2 [28]) and those with multiple defects (presumed fixed pseudogenes). [file 1741-7007-6-42-S9.jpeg]

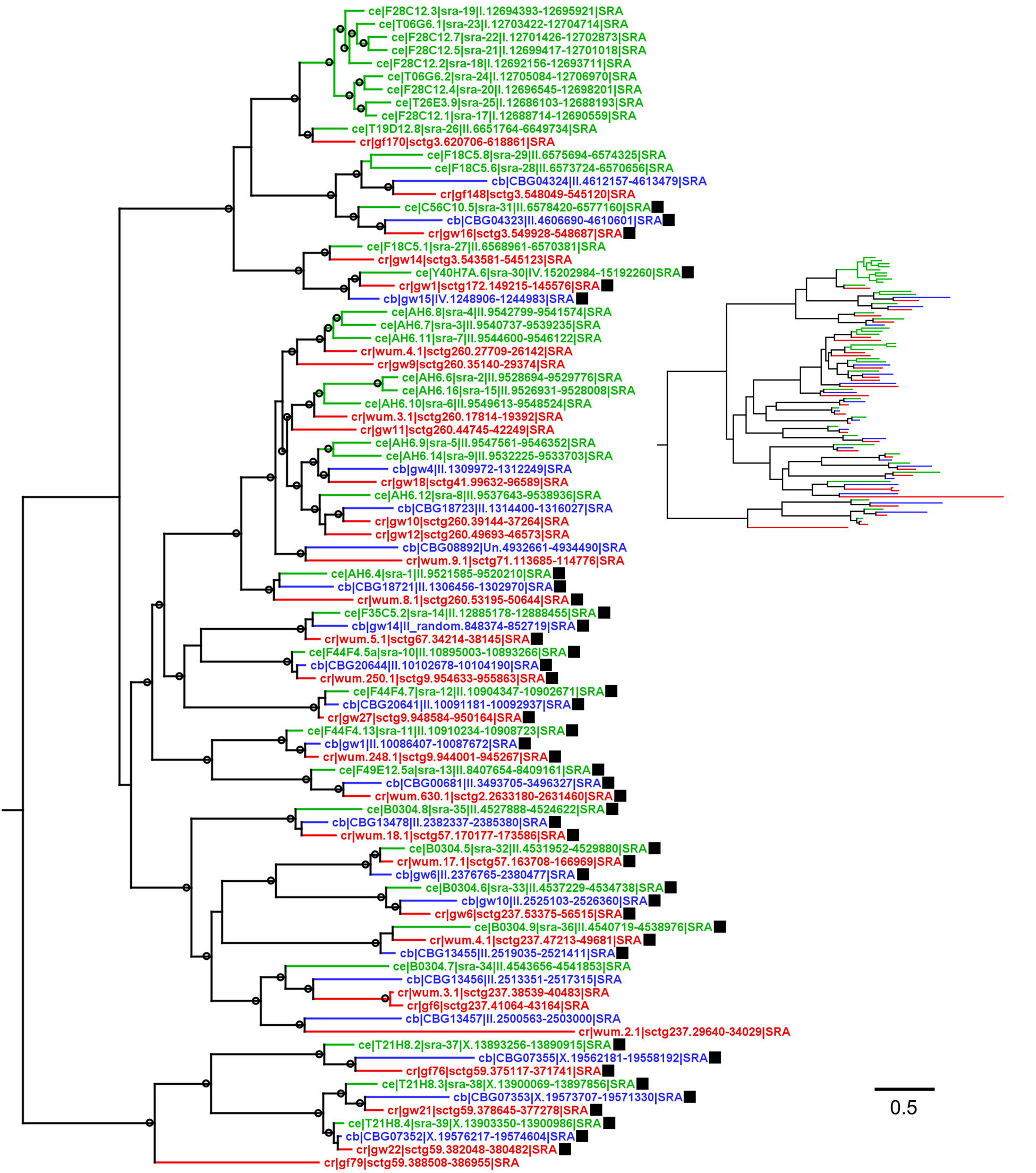

Supplement: Additional file 10 — Table presenting the summary of evolutionary properties of chemoreceptor genes from Caenorhabditis elegans, C. briggsae, and C. remanei. Metabotropic neurotransmitter and FRMF-amide receptor related genes are also shown for comparison. Fraction strict orthologs is the fraction of C. elegans genes with single orthologs in both C. briggsae and C. remanei, as determined the protein tree. Fraction clustered in the genome is the fraction of C. elegans genes that have another family member located within five genes in the genome. Tree gene number indicates the number of genes used for protein tree analysis (see Methods for specifics). For the C. elegans tree gene number column, the number in parentheses is the number of genes predicted to encode functional receptors in the reference N2 genome. Naively, we expect that a similar fraction of genes from the other two species will be functional in their respective reference genomes. For example, in the srh family there will be (218/294) × 214 functional genes in C. remanei and (218/294) × 165 functional genes in C. briggsae. [file 1741-7007-6-42-S10.jpeg]

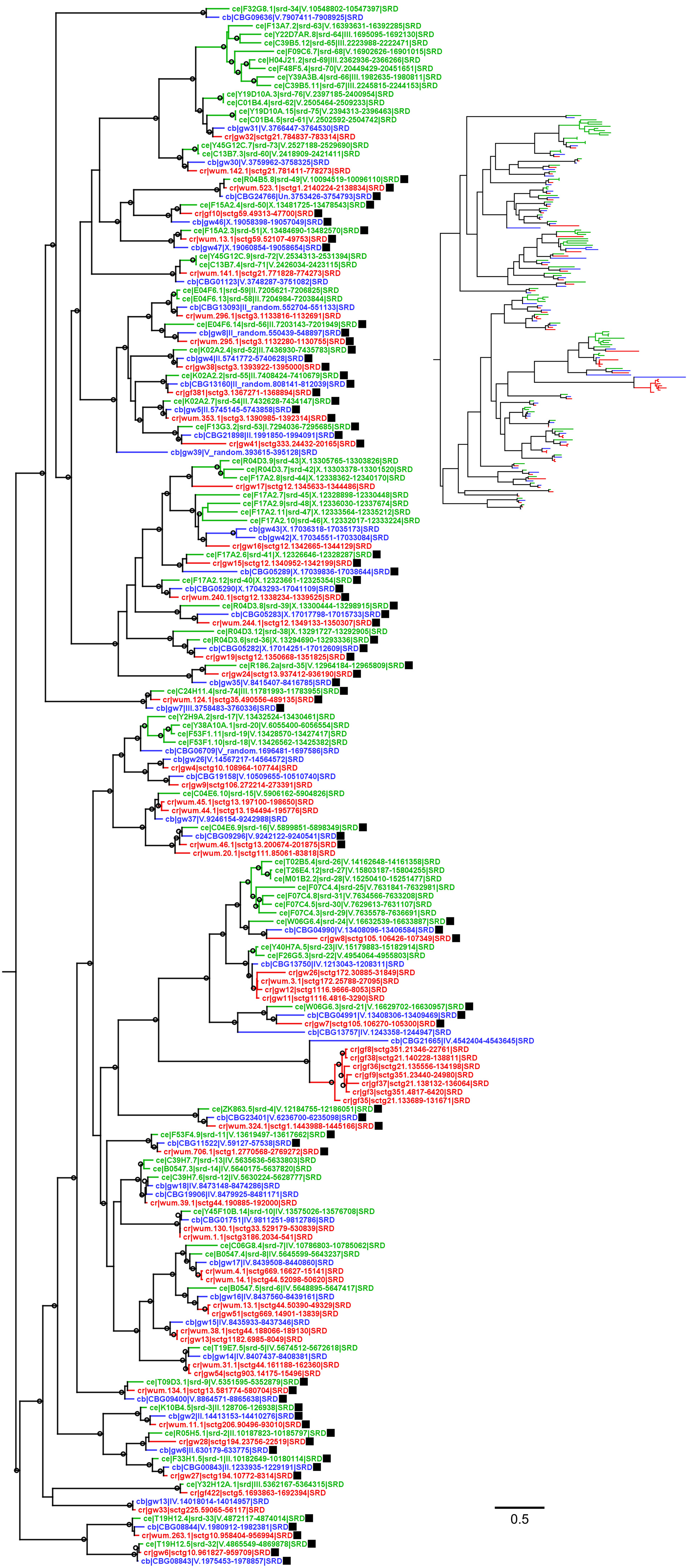

Supplement: Additional file 14 — Maximum-likelihood tree of SRA proteins.Caenorhabditis elegans names are green, C. briggsae names are blue, and C. remanei names are red. Species-specific clades are emphasized by having their branch lines match the species color. The smaller inset is the same tree with names removed, which shows the tree structure more clearly. Open circles on branches indicate a branch support value of 0.9 or higher, as computed by phyml-alrt. Strict ortholog trios (1-1-1) are marked with a filled black square. The tree was rooted by inclusion of a sampling of SRAB proteins (not shown). The scale bar indicates number of amino acid changes per site in the large tree. Each name includes a species identifier, gene identifiers, and genome start and end coordinates for the corresponding gene model. The C. elegans gene names include both a standard genome project name (for example, F28C12.3) and a genetic gene name (for example, sra-19). The C. briggsae gene name is the brigpep WormBase name when applicable (for example, CBG04324) or an arbitrarily numbered GeneWise prediction number (for example, gw15). The C. remanei names are either the WormBase wum gene prediction (for example, wum.4.1), the WormBase genefinder prediction (for example, gf170), or an arbitrarily numbered GeneWise prediction number (for example, gw16). The wum or gf names combined with the supercontig number uniquely identify the prediction in the current C. remanei prediction set on WormBase. The sequences analyzed are given in Additional files 11 to 13. All trees are available in Newick format upon request. [file 1741-7007-6-42-S14.jpeg]

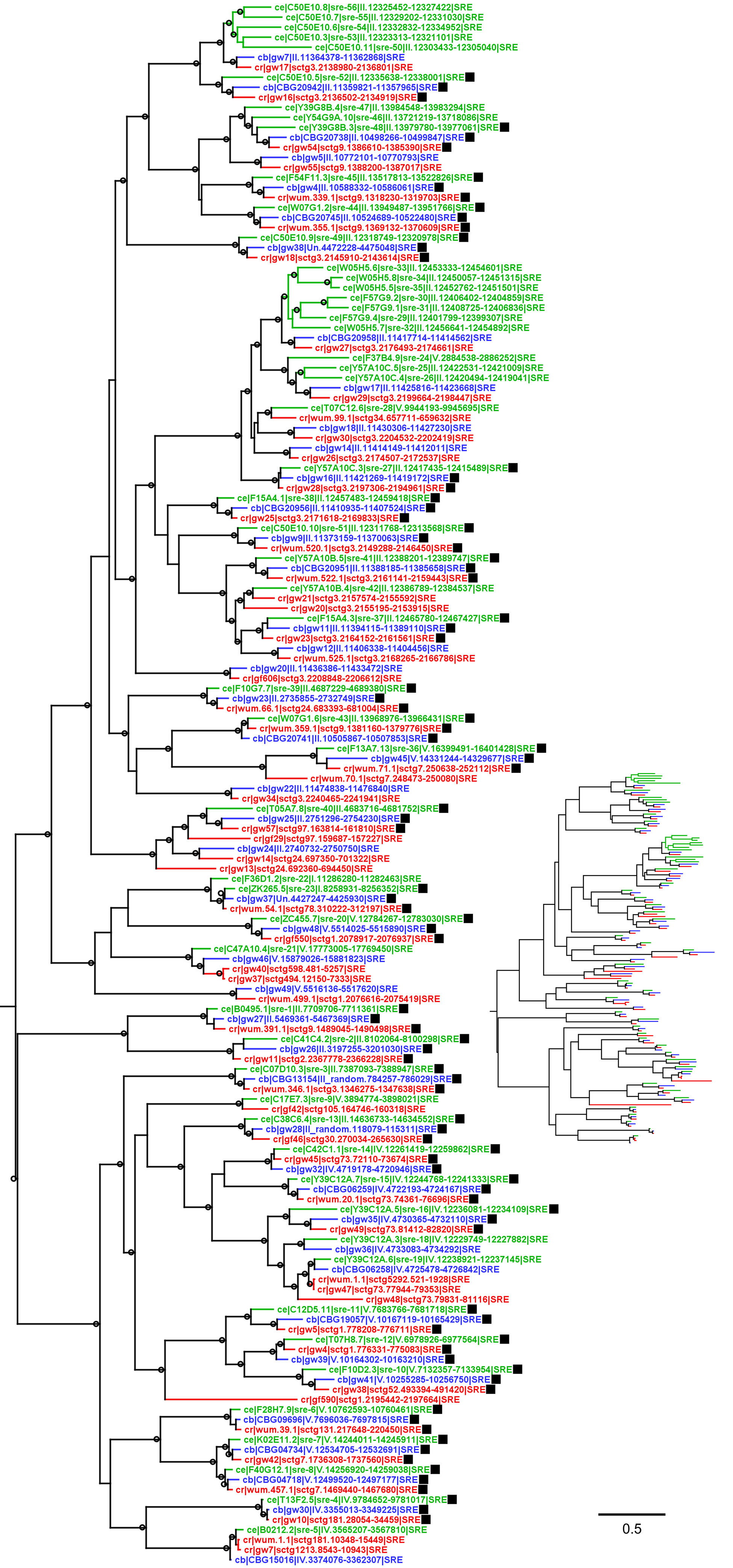

Supplement: Additional file 15 — Maximum-likelihood tree of SRAB proteins. See Additional file 14 for the legend. The tree was rooted by inclusion of a sampling of SRA proteins (not shown). [file 1741-7007-6-42-S15.jpeg]

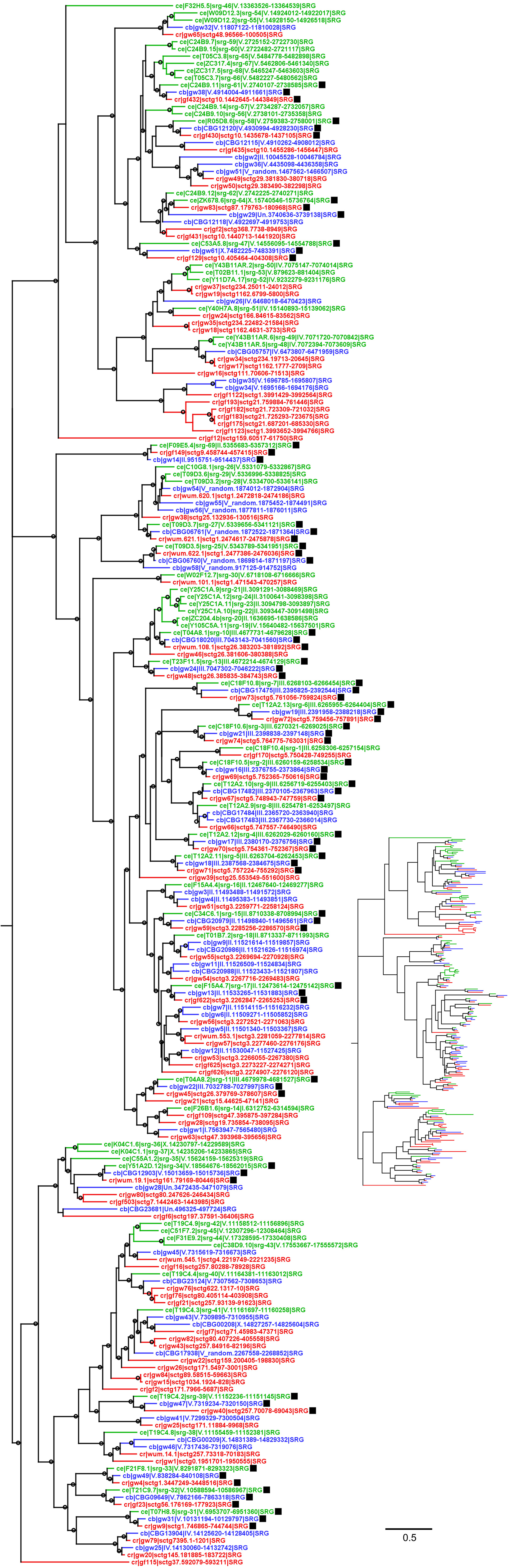

Supplement: Additional file 16 — Maximum-likelihood tree of SRB proteins. See Additional file 14 for the legend. The tree was rooted by inclusion of a sampling of SRAB proteins (not shown). [file 1741-7007-6-42-S16.jpeg]

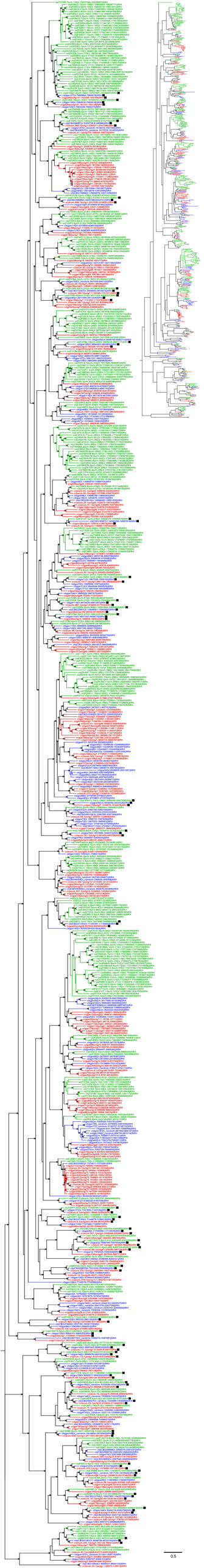

Supplement: Additional file 17 — Maximum-likelihood tree of SRBC proteins. See Additional file 14 for the legend. The tree is unrooted. [file 1741-7007-6-42-S17.jpeg]

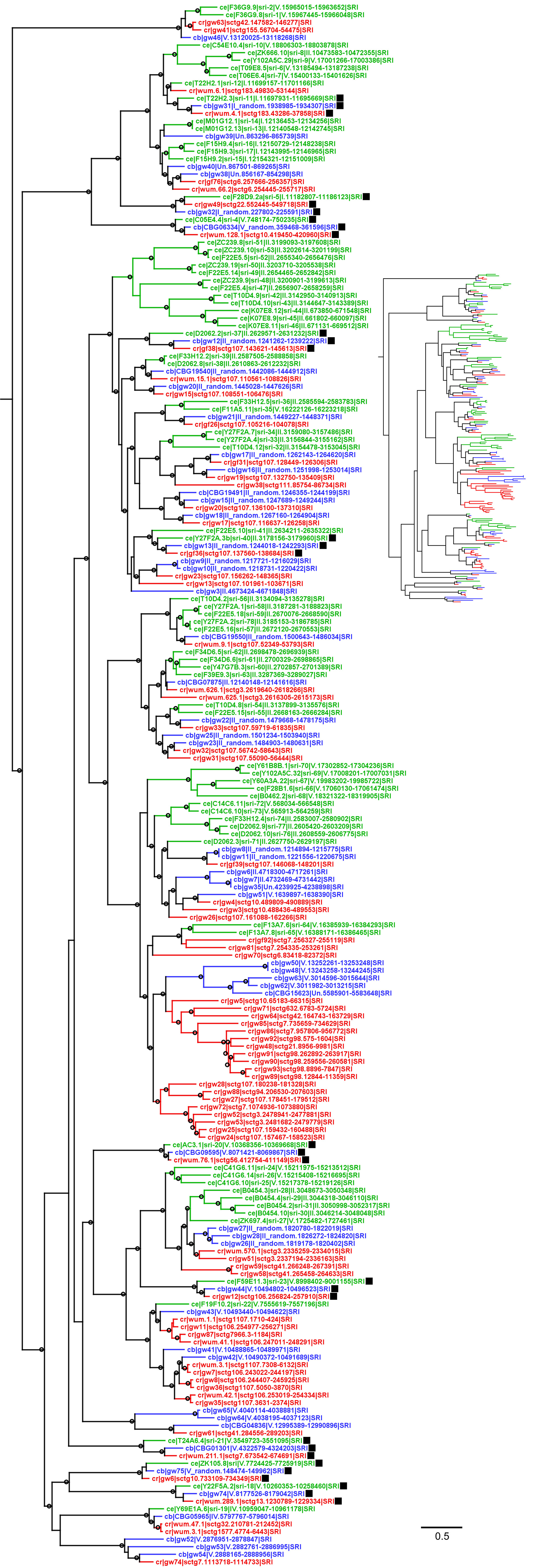

Supplement: Additional file 18 — Maximum-likelihood tree of SRD proteins. See Additional file 14 for the legend. The tree was rooted by inclusion of a sampling of STR proteins (not shown). [file 1741-7007-6-42-S18.jpeg]

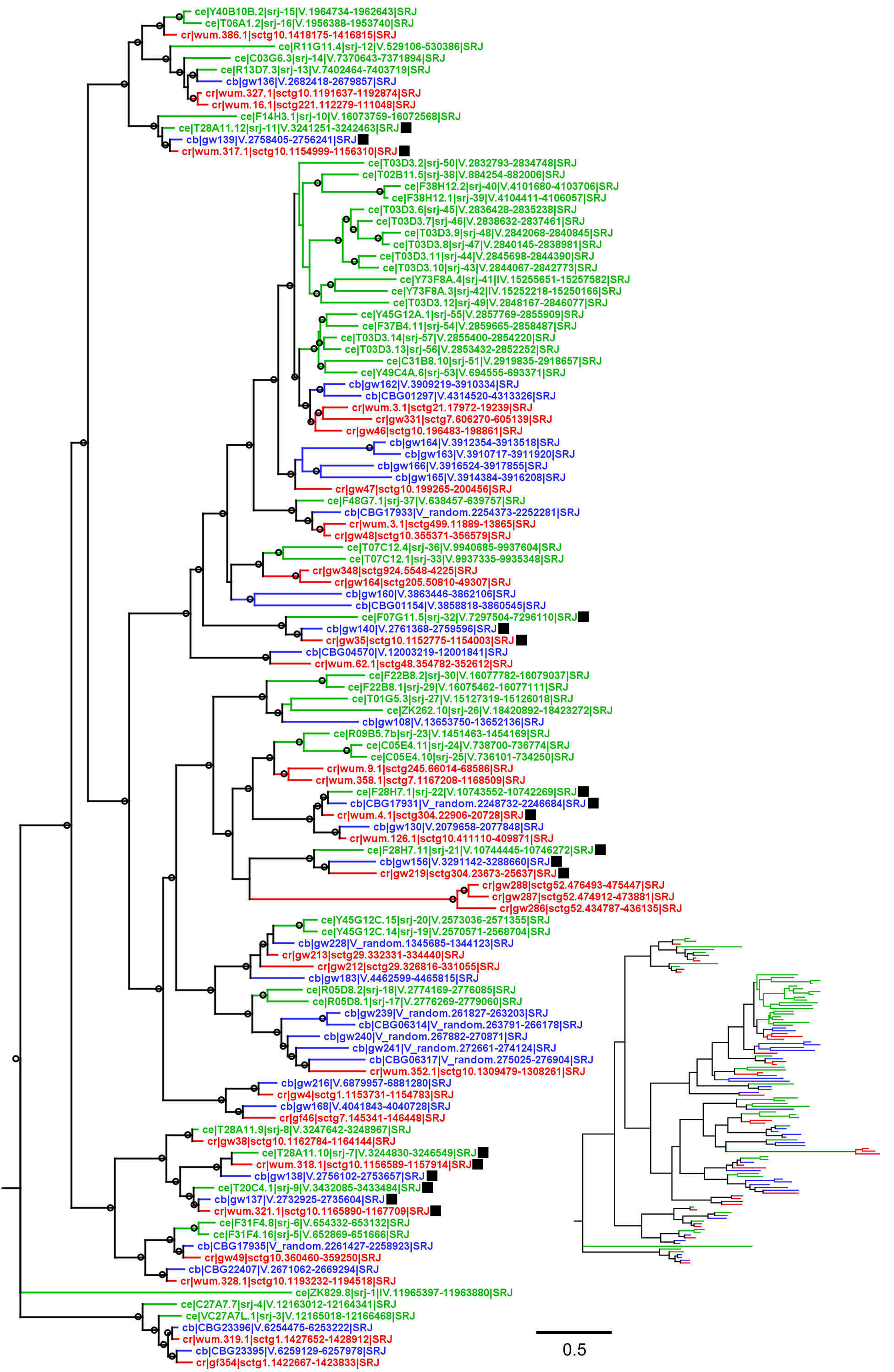

Supplement: Additional file 19 — Maximum-likelihood tree of SRE proteins. See Additional file 14 for the legend. The tree was rooted by inclusion of a sampling of SRA proteins (not shown). [file 1741-7007-6-42-S19.jpeg]

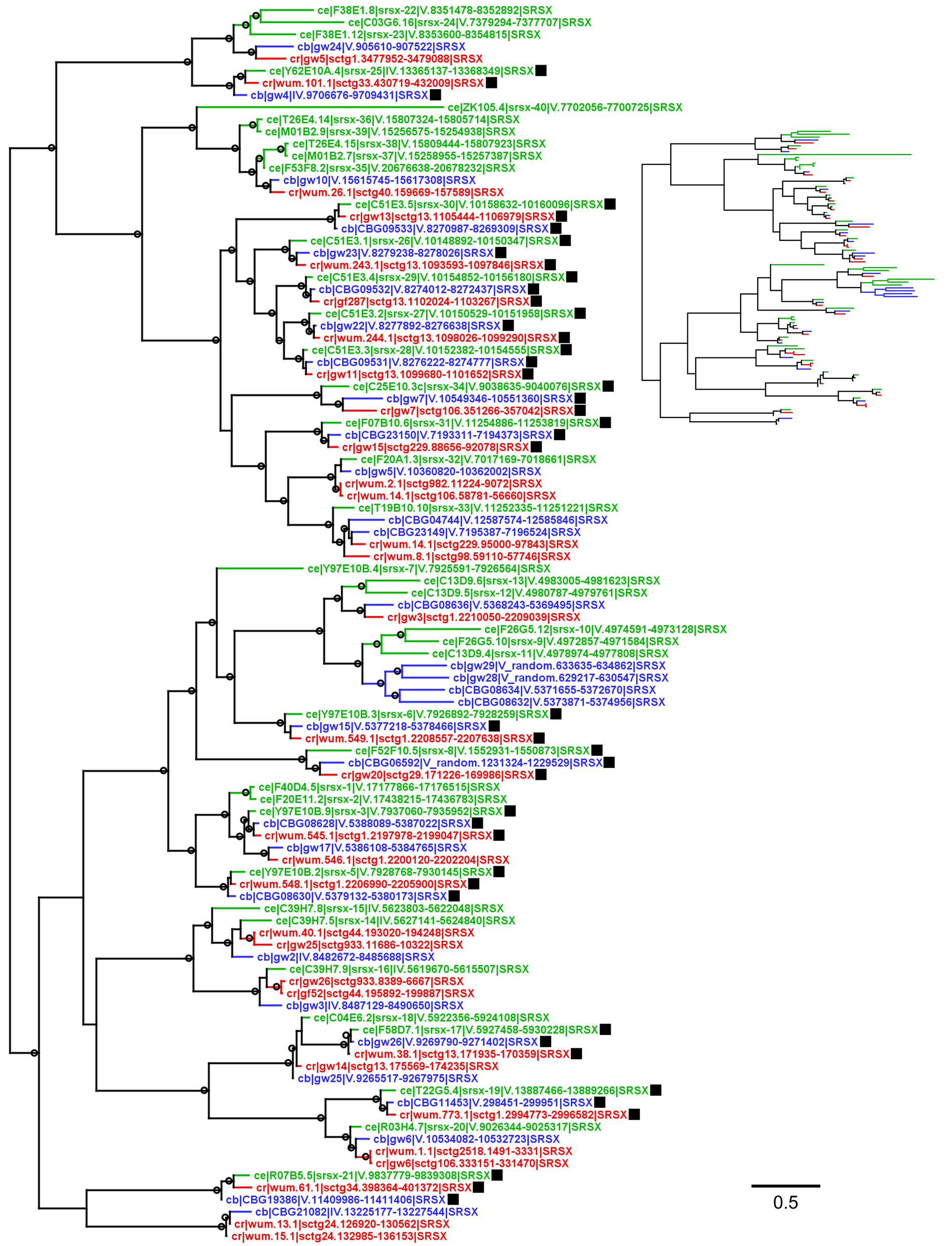

Supplement: Additional file 20 — Maximum-likelihood tree of SRG proteins. See Additional file 14 for the legend. The tree was rooted by inclusion of a sampling of SRU proteins (not shown). [file 1741-7007-6-42-S20.jpeg]

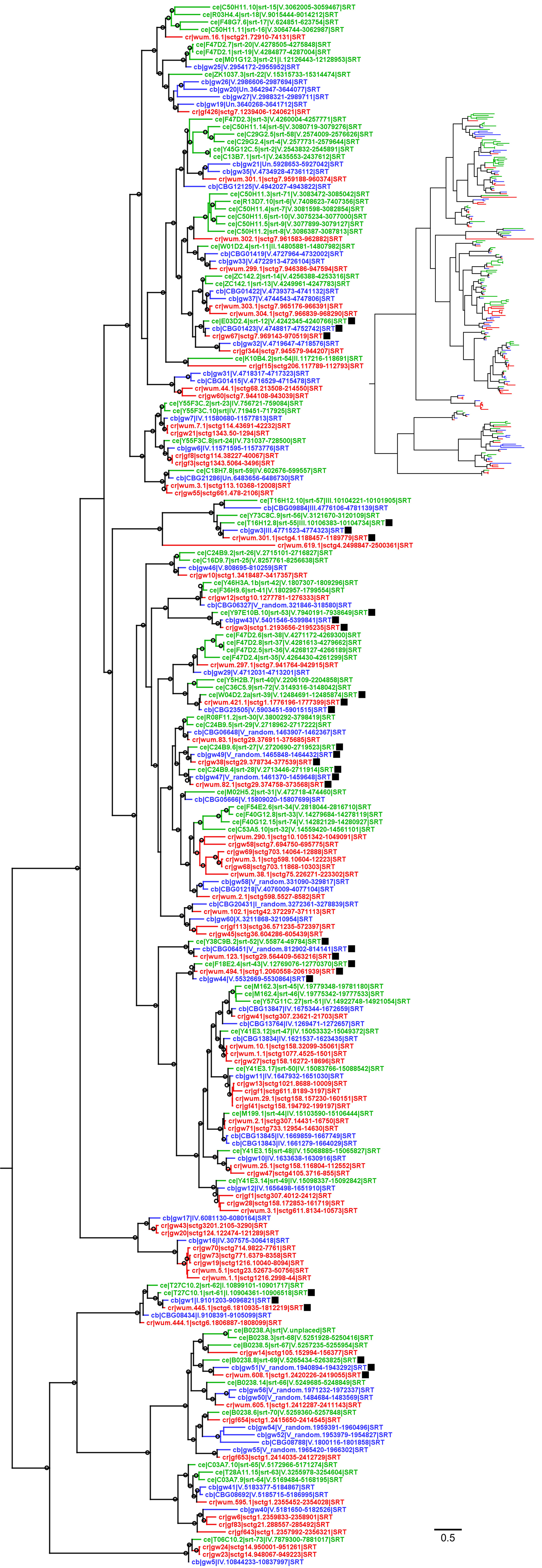

Supplement: Additional file 21 — Maximum-likelihood tree of SRH proteins. See Additional file 14 for the legend. The tree was rooted by inclusion of a sampling of SRI proteins (not shown). [file 1741-7007-6-42-S21.jpeg]

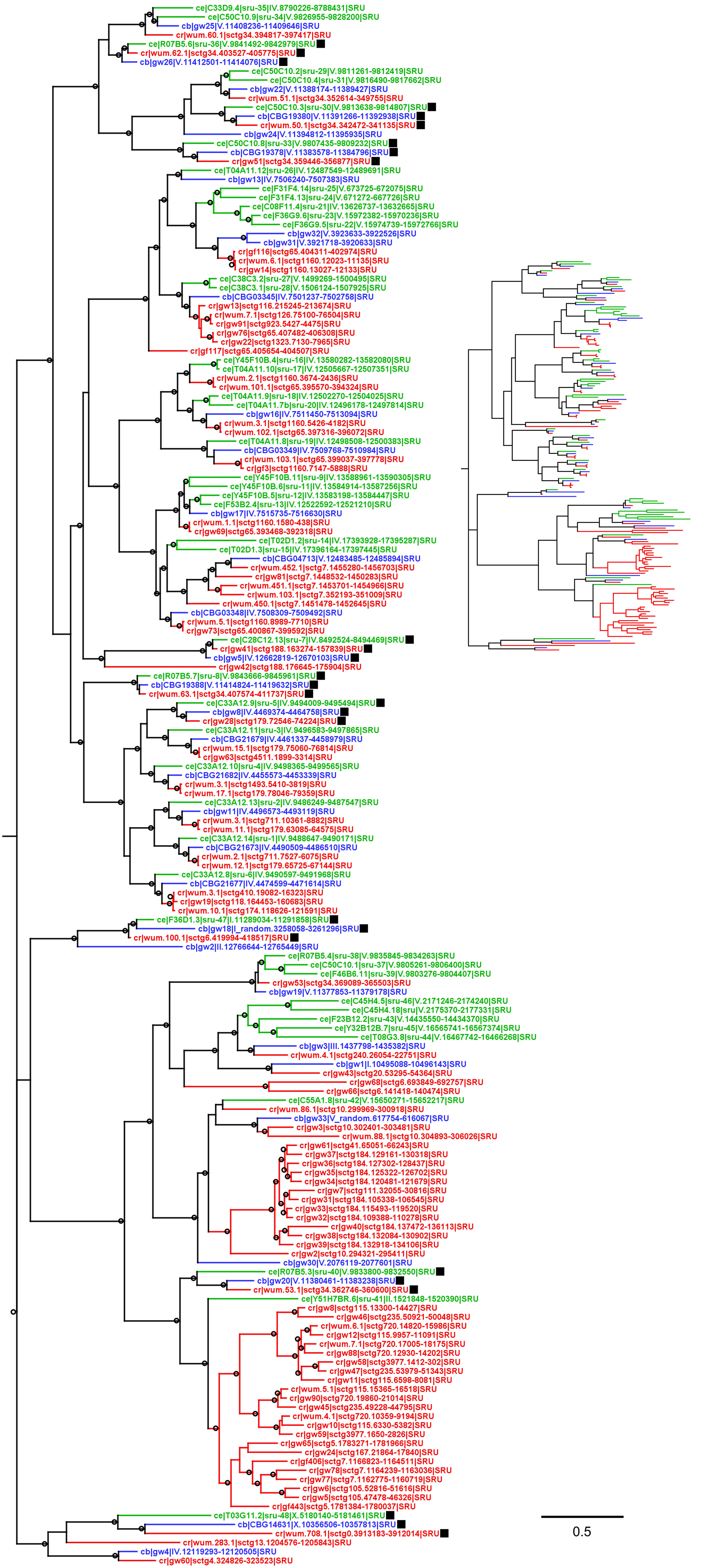

Supplement: Additional file 22 — Maximum-likelihood tree of SRI proteins. See Additional file 14 for the legend. The tree was rooted by inclusion of a sampling of SRH proteins (not shown). [file 1741-7007-6-42-S22.jpeg]

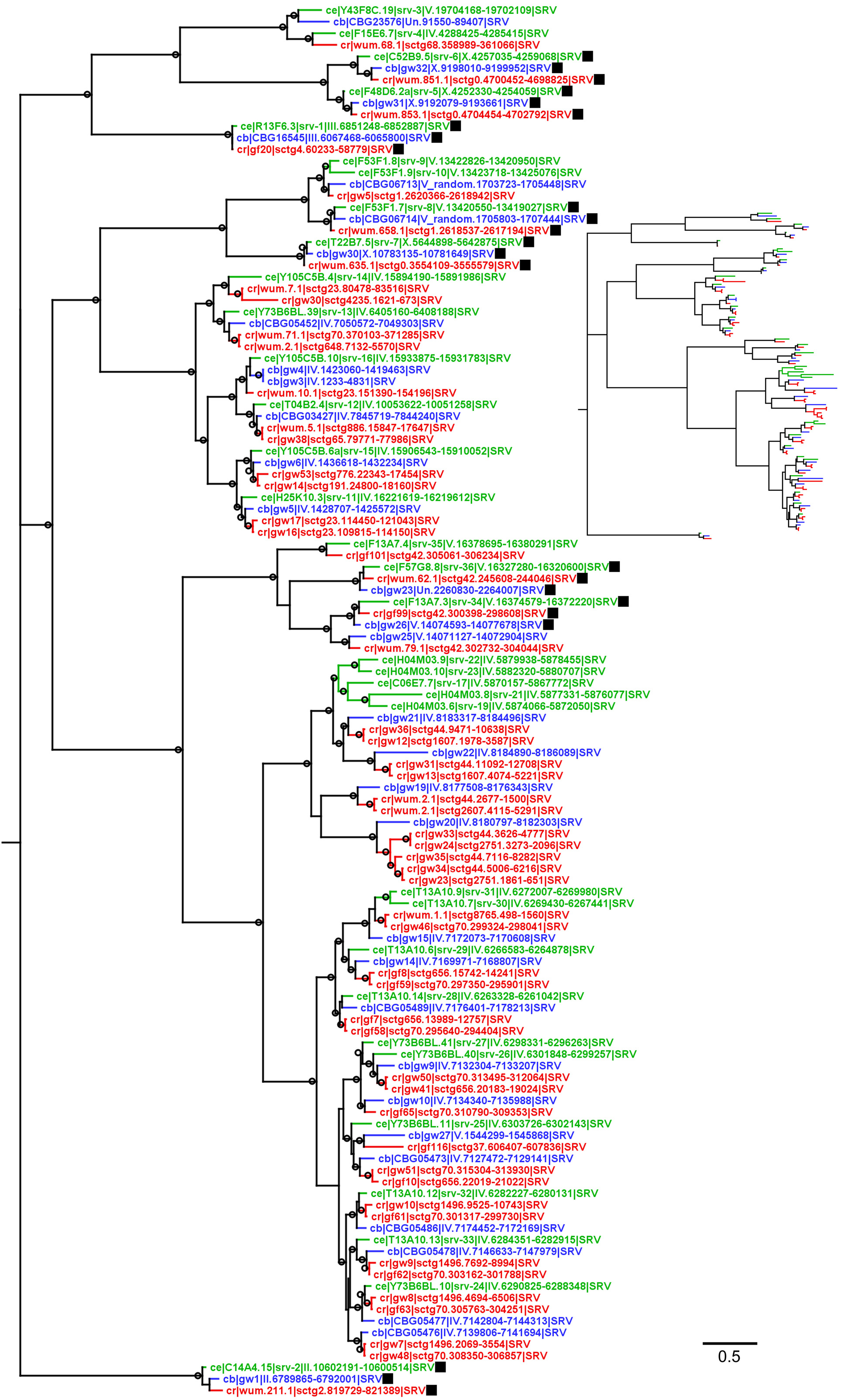

Supplement: Additional file 23 — Maximum-likelihood tree of SRJ proteins. See Additional file 14 for the legend. The tree was rooted by inclusion of a sampling of STR proteins (not shown). [file 1741-7007-6-42-S23.jpeg]

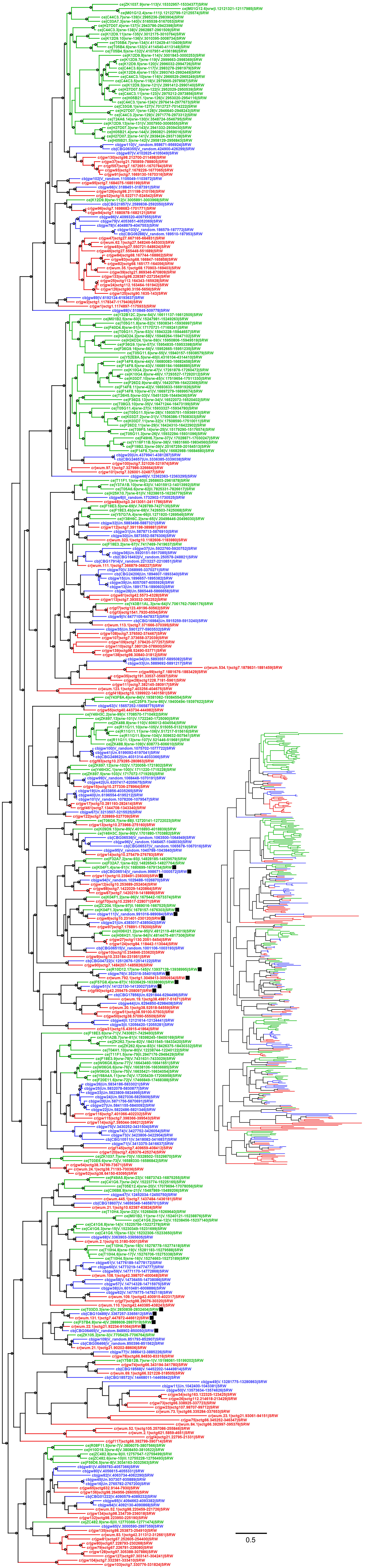

Supplement: Additional file 24 — Maximum-likelihood tree of SRSX proteins. See Additional file 14 for the legend. The tree is unrooted. [file 1741-7007-6-42-S24.jpeg]

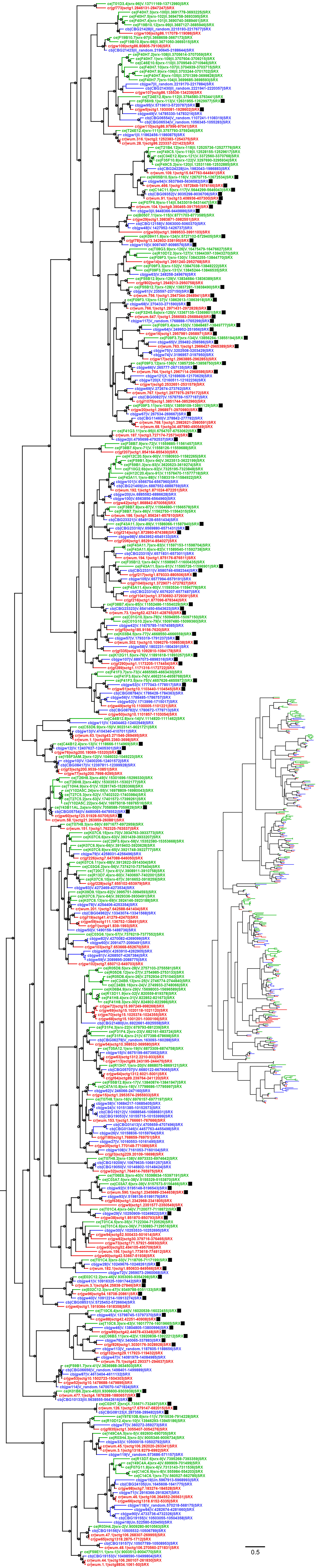

Supplement: Additional file 25 — Maximum-likelihood tree of SRT proteins. See Additional file 14 for the legend. The tree was rooted by inclusion of a sampling of SRX proteins (not shown). [file 1741-7007-6-42-S25.jpeg]

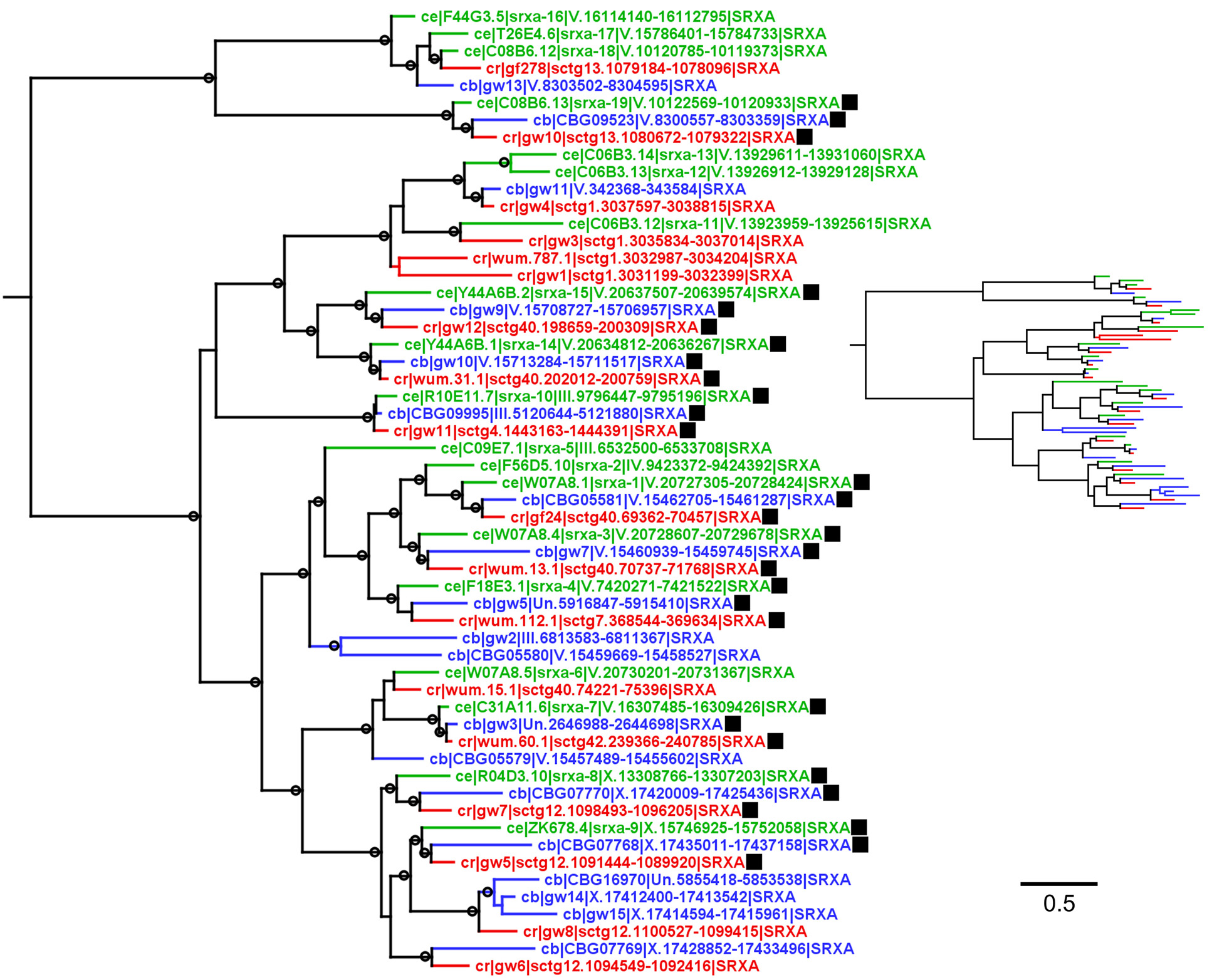

Supplement: Additional file 26 — Maximum-likelihood tree of SRU proteins. See Additional file 14 for the legend. The tree was rooted by inclusion of a sampling of SRV proteins (not shown). [file 1741-7007-6-42-S26.jpeg]

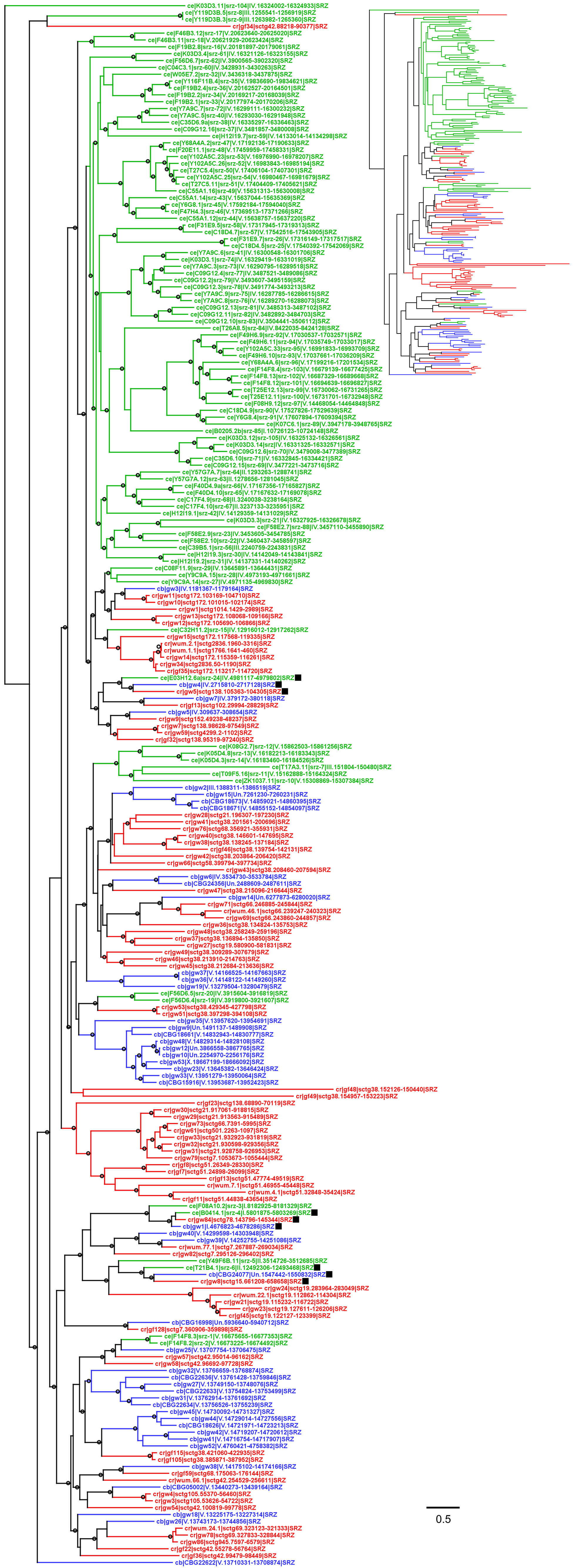

Supplement: Additional file 27 — Maximum-likelihood tree of SRV proteins. See Additional file 14 for the legend. The tree was rooted by inclusion of a sampling of SRU proteins (not shown). [file 1741-7007-6-42-S27.jpeg]

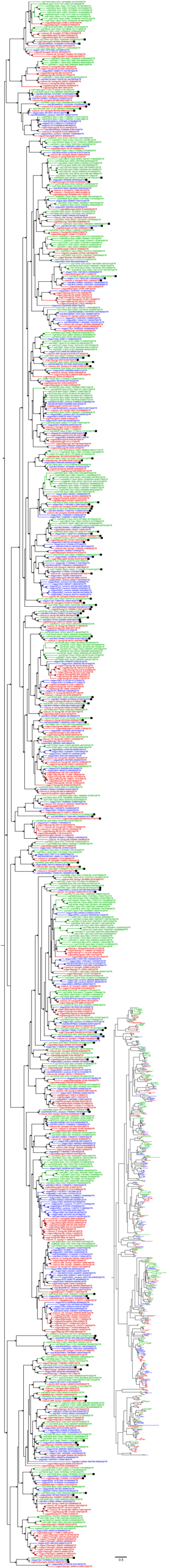

Supplement: Additional file 28 — Maximum-likelihood tree of SRW proteins. See Additional file 14 for the legend. The tree is unrooted. [file 1741-7007-6-42-S28.jpeg]

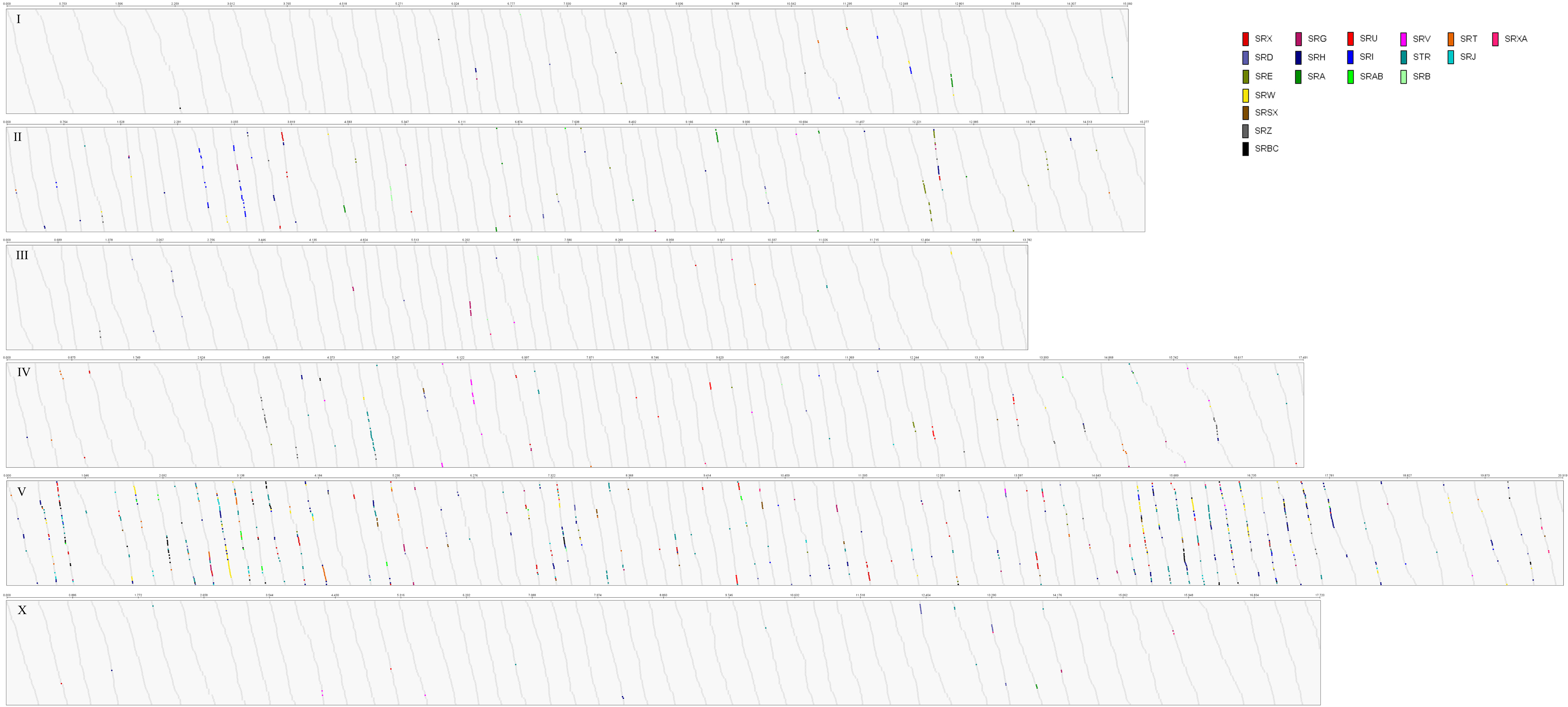

Supplement: Additional file 29 — Maximum-likelihood tree of SRX proteins. See Additional file 14 for the legend. The tree was rooted by inclusion of a sampling of SRT proteins (not shown). [file 1741-7007-6-42-S29.jpeg]

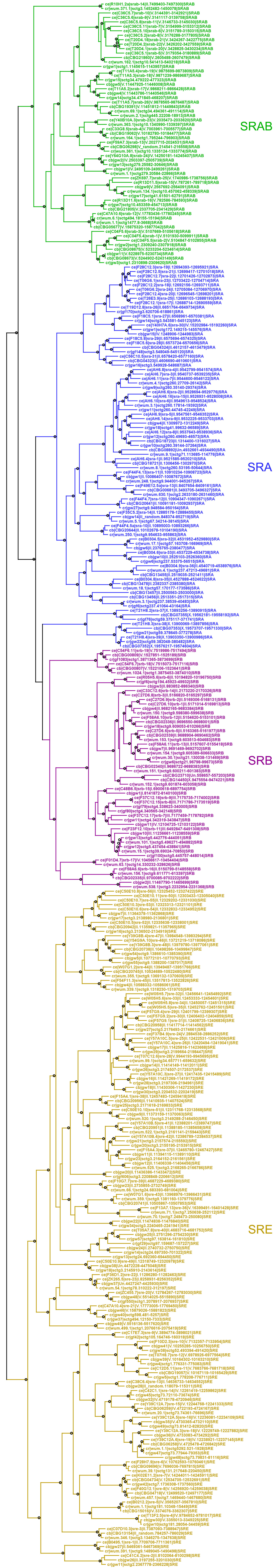

Supplement: Additional file 30 — Maximum-likelihood tree of SRXA proteins. See Additional file 14 for the legend. The tree was rooted by inclusion of a sampling of SRV proteins (not shown). [file 1741-7007-6-42-S30.jpeg]

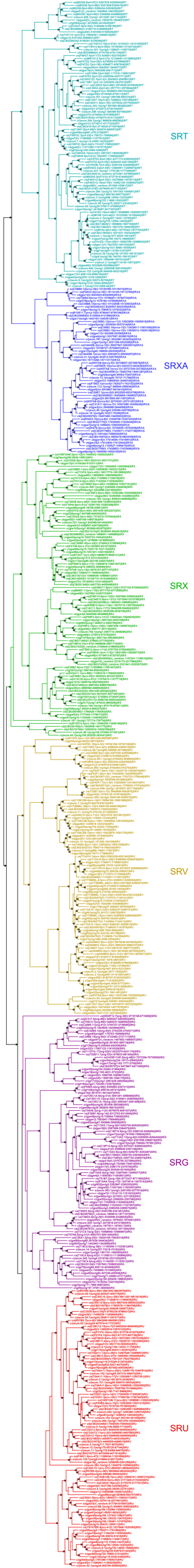

Supplement: Additional file 31 — Maximum-likelihood tree of SRZ proteins. See Additional file 14 for the legend. The tree is unrooted. [file 1741-7007-6-42-S31.jpeg]

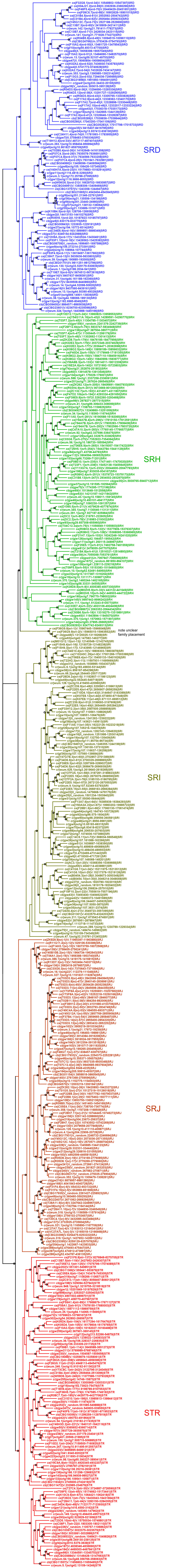

Supplement: Additional file 32 — Maximum-likelihood tree of STR proteins. See Additional file 14 for the legend. The tree was rooted by inclusion of a sampling of SRJ proteins (not shown). [file 1741-7007-6-42-S32.jpeg]

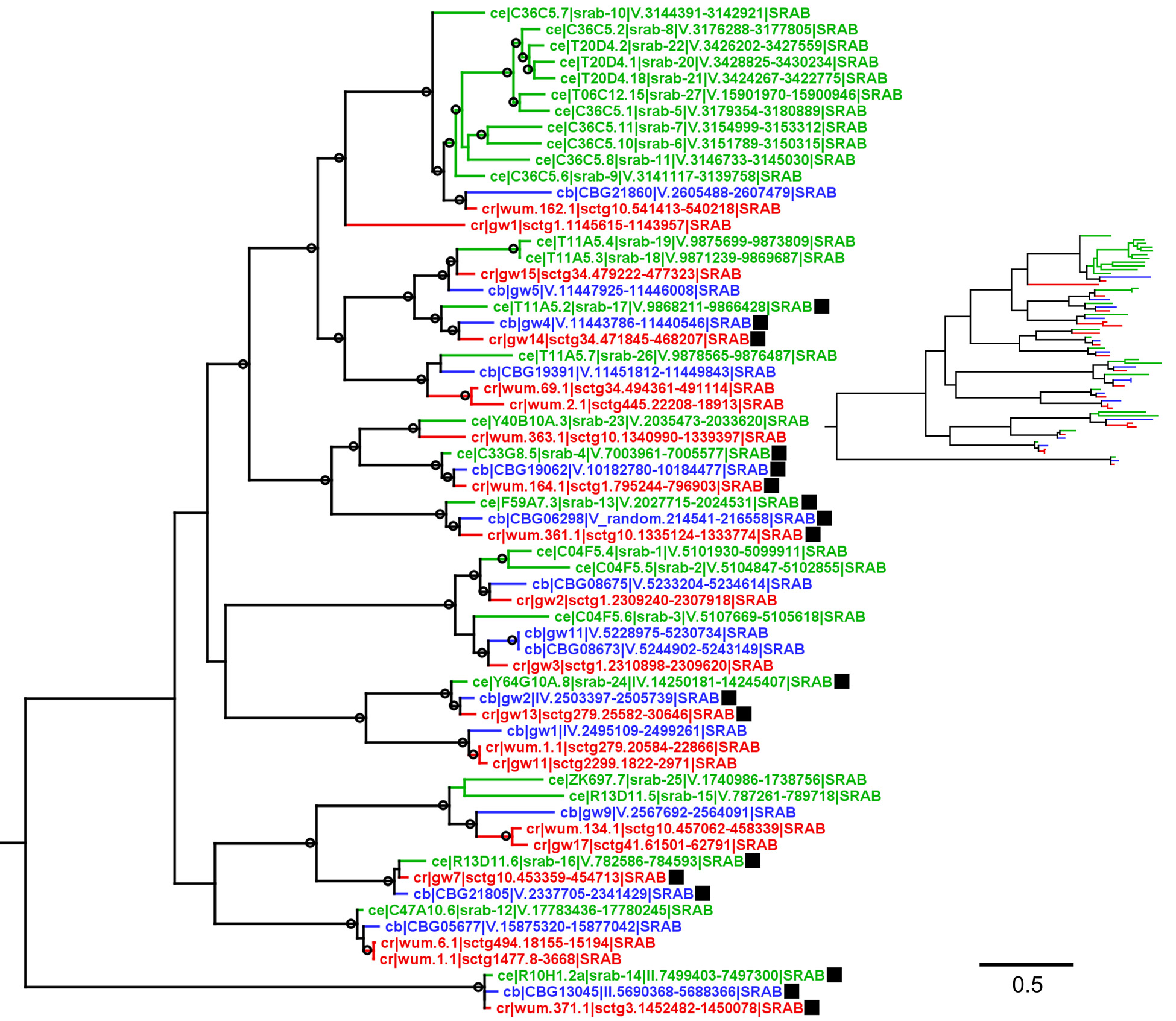

Supplement: Additional file 11 — All Caenorhabditis elegans proteins used in protein tree analysis. The genome start and end position and family are given as part of the fasta name. Coding strand is implied by the order of the two genome coordinates. The list includes some possibly defective proteins if they met our criteria for inclusion in tree analysis (see Methods). [file 1741-7007-6-42-S11.jpeg]

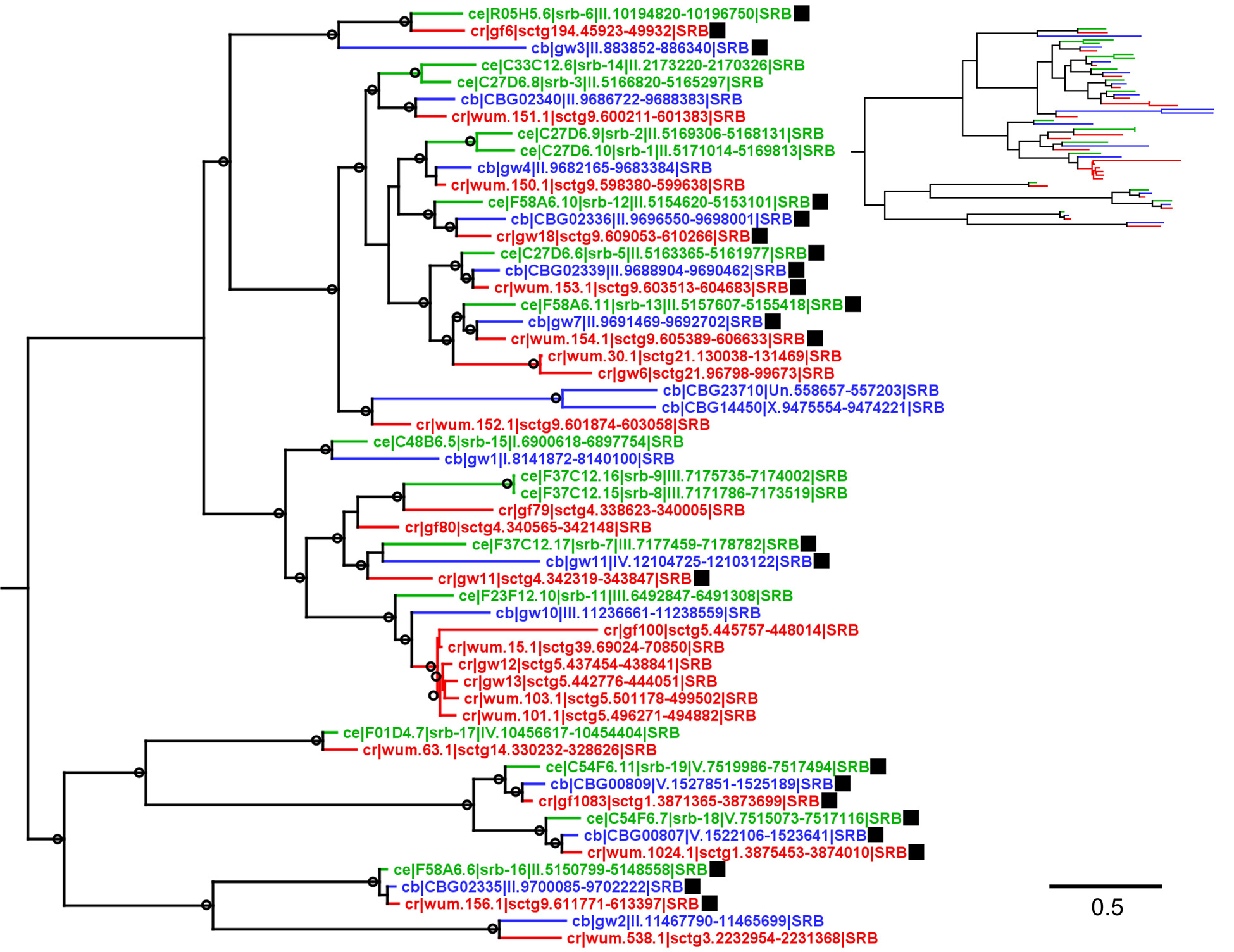

Supplement: Additional file 12 — All Caenorhabditis briggsae proteins used in protein tree analysis. The second field indicates the WormBase gene identifier or an arbitrary GeneWise number (see Methods). See also Additional file 11. [file 1741-7007-6-42-S12.jpeg]

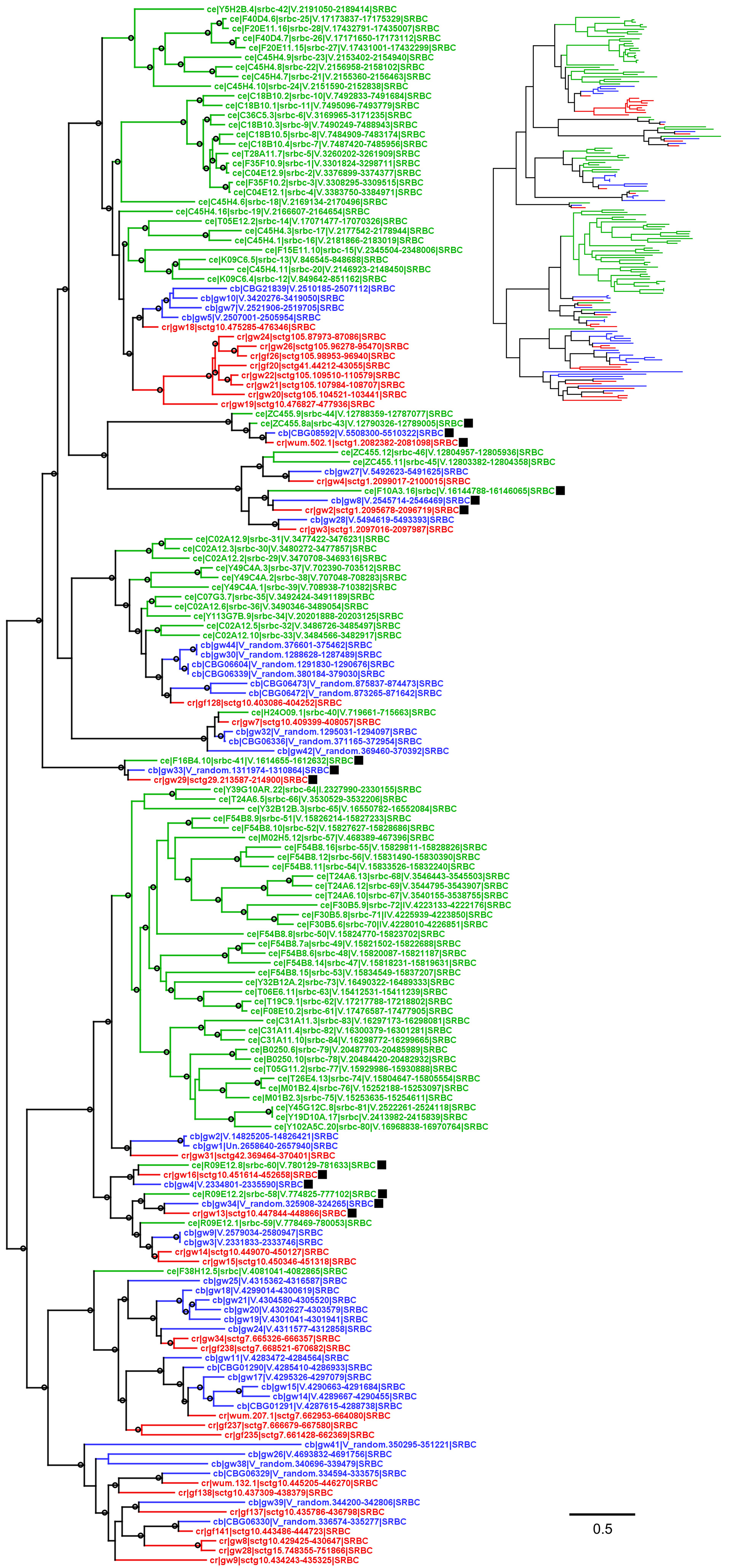

Supplement: Additional file 13 — All Caenorhabditis remanei proteins used in protein tree analysis. The second field indicates the wum gene identifier, the genefinder identifier, or an arbitrary GeneWise number (see Methods). See also Additional file 11. [file 1741-7007-6-42-S13.jpeg]
